# Distinct transcriptional alterations distinguish Lewy body disease from Alzheimer’s disease

**DOI:** 10.1093/brain/awae202

**Published:** 2024-06-25

**Authors:** Kimberly C Olney, Benjamin E Rabichow, Aleksandra M Wojtas, Michael DeTure, Pamela J McLean, Dennis W Dickson, Rui Chang, Owen A Ross, John D Fryer

**Affiliations:** Department of Neuroscience, Mayo Clinic, Scottsdale, AZ 85259, USA; Department of Neuroscience, Mayo Clinic, Scottsdale, AZ 85259, USA; Program in Neuroscience, Mayo Clinic, Scottsdale, AZ 85259, USA; Department of Neuroscience, Mayo Clinic, Scottsdale, AZ 85259, USA; Program in Neuroscience, Mayo Clinic, Scottsdale, AZ 85259, USA; Department of Neuroscience, Mayo Clinic, Jacksonville, FL 32224, USA; Department of Neuroscience, Mayo Clinic, Jacksonville, FL 32224, USA; Department of Neuroscience, Mayo Clinic, Jacksonville, FL 32224, USA; Department of Neurology, University of Arizona, Tucson, AZ 85724, USA; Department of Neuroscience, Mayo Clinic, Jacksonville, FL 32224, USA; Department of Neuroscience, Mayo Clinic, Scottsdale, AZ 85259, USA; Program in Neuroscience, Mayo Clinic, Scottsdale, AZ 85259, USA

**Keywords:** neuropathologies, dementia, bulk RNAseq, transcriptome, WGCNA

## Abstract

Lewy body dementia and Alzheimer's disease (AD) are leading causes of cognitive impairment, characterized by distinct but overlapping neuropathological hallmarks. Lewy body disease (LBD) is characterized by α-synuclein aggregates in the form of Lewy bodies as well as the deposition of extracellular amyloid plaques, with many cases also exhibiting neurofibrillary tangle (NFT) pathology. In contrast, AD is characterized by amyloid plaques and neurofibrillary tangles. Both conditions often co-occur with additional neuropathological changes, such as vascular disease and TDP-43 pathology.

To elucidate shared and distinct molecular signatures underlying these mixed neuropathologies, we extensively analysed transcriptional changes in the anterior cingulate cortex, a brain region critically involved in cognitive processes. We performed bulk tissue RNA sequencing from the anterior cingulate cortex and determined differentially expressed genes (*q-*value <0.05) in control (*n* = 81), LBD (*n* = 436), AD (*n* = 53) and pathological amyloid cases consisting of amyloid pathology with minimal or no tau pathology (*n* = 39). We used gene set enrichment and weighted gene correlation network analysis to understand the pathways associated with each neuropathologically defined group.

LBD cases had strong upregulation of inflammatory pathways and downregulation of metabolic pathways. The LBD cases were further subdivided into either high Thal amyloid, Braak NFT, or low pathological burden cohorts. Compared to the control cases, the LBD cohorts consistently showed upregulation for genes involved in protein folding and cytokine immune response, as well as downregulation of fatty acid metabolism. Surprisingly, concomitant tau pathology within the LBD cases resulted in no additional changes. Some core inflammatory pathways were shared between AD and LBD but with numerous disease-specific changes. Direct comparison of LBD cohorts versus AD cases revealed strong enrichment of synaptic signalling, behaviour and neuronal system pathways. Females had a stronger response overall in both LBD and AD, with several sex-specific changes.

Overall, the results identify genes commonly and uniquely dysregulated in neuropathologically defined LBD and AD cases, shedding light on shared and distinct molecular pathways. Additionally, the study underscores the importance of considering sex-specific changes in understanding the complex transcriptional landscape of these neurodegenerative diseases.

## Teaser

Olney *et al.* analyse the transcriptome of individuals with Lewy body disease and Alzheimer's disease, identifying upregulation of neuroinflammatory pathways in both disease types. While some core inflammatory pathways were altered in both diseases, numerous disease-specific changes were also observed.

## Introduction

Lewy body dementia is a common cause of dementia and is characterized pathologically by the hallmark accumulation of cortical and subcortical intraneuronal α-synuclein rich deposits called Lewy bodies (LB).^1^ Individuals with Lewy body dementia typically have other concomitant brain pathologies, including extracellular amyloid-β (Aβ) plaques and sometimes neurofibrillary tangles of tau protein.^2-5^ Clinically, Lewy body dementia can be diagnosed as either Parkinson's disease dementia (PDD) or dementia with Lewy bodies (DLB), depending on the temporal sequence of motor and cognitive symptom presentation.^4,6^ Ultimately, Lewy body dementia pathogenesis causes progressive motor and cognitive deficits, likely driven by the degeneration of nigro-striatal circuitry and widespread cortical atrophy, respectively.^4,7,8^ Although patients with Lewy body dementia display unique phenotypes—the confluence of plaque and Lewy pathology, fluctuating cognition and visual hallucinations—it has clinicopathological overlap with Alzheimer's (AD) and Parkinson's disease (PD). Therefore, there is a critical need to elucidate the molecular alterations in the brain caused by Lewy body pathology and determine which alterations are exclusive to dementia with Lewy bodies or other neurodegenerative diseases.

Several studies have examined gene expression changes in Lewy body dementia,^9,10^ as reviewed by Chowdhury and Rajkumar^11^; however, these studies were underpowered to determine sex-specific transcriptional alterations or lacked a comparison to other neurodegenerative diseases. This study focuses on a well-characterized neuropathologic cohort, including 436 Lewy body disease (LBD) cases, 53 AD cases, 39 cases of pathological amyloid (predominant amyloid only pathology) and 81 age-matched controls. Our objective was to uncover shared and distinct molecular changes that occur in LBD compared to AD to better understand the pathological drivers that underpin these distinct diseases.

LBD is subdivided into three main subtypes according to the distribution pattern of Lewy body pathology: brainstem-restricted (BLBD) through transitional (TLBD) and ultimately diffuse (DLBD), in which Lewy bodies occur throughout the brain, including the outer cortices.^12,13^ LBD is primarily characterized by the presence of α-synuclein aggregates,^1^ whereas tau pathology resulting in the abnormal accumulation and aggregation of the tau protein within neurons is largely associated with AD.^14^ While tau pathology is closely linked to AD, there can be overlap in neuropathological features among different neurodegenerative conditions.^2-5,14^*APOE* ε4 positive individuals are at greater risk of developing clinical Lewy body dementia as well as AD.^15^ Because of the pathological heterogeneity, we included further subdivision of our LBD cases into cohorts exhibiting primarily LB/synuclein (S) pathology (*n* = 95), LB pathology accompanied by high Thal amyloid phase but low Braak NFT stage (AS; *n* = 161) and LB pathology coupled with high Thal amyloid and high Braak NFT stage (ATS; *n* = 178).

Sex differences manifest across various neurodegenerative conditions, including Lewy body dementia, where unique risk factors, symptoms and progression patterns emerge between females and males.^16-18^ Here, sex was examined jointly and separately to offer insights into sex-shared and sex-specific transcriptional changes in AD and LBD. In summary, we utilized a variety of analytical approaches, encompassing differential expression and co-expression analyses, to discern genes and pathways linked to each neuropathologically defined disease, both overall and stratified by sex.

## Materials and methods

### Sample selection and classification

The Mayo Clinic Florida brain bank is a large tissue repository with over 12 000 brains. LBD cases were selected based on available tissue and the presence of LB, leading to a high or intermediate likelihood of clinical Lewy body dementia, as defined by the McKeith criteria.^2,3^ Comparison groups were selected based on tissue availability and attempts to broadly age and sex match. Clinical diagnoses of dementia within the brain bank database are not always available and were not performed by the same neurologist; however, pathology annotation for each sample was curated by a single neuropathologist (D.W.D.) at the Mayo Clinic. Cause of death and post-mortem interval are not available for some of the cases. The presence of age-related tau astrogliopathy is not routinely screened by D.W.D. and thus was not assessed. Cases in this study were excluded if there was any other significant pathology that would alter the pathologic diagnoses made by D.W.D. Anterior cingulate cortex tissue samples from the Mayo Clinic brain bank were collected for 609 individuals. Control samples have a Braak NFT stage of less than III and Thal amyloid phase of less than 2, and pathological amyloid (PA) is defined as Braak NFT stage of less than III and Thal amyloid phase of greater than or equal to 2 (Table 1). AD is defined as Thal amyloid phase of equal or greater than 3 and a Braak NFT stage of V and greater. A sample was categorized as LBD if LBs were present. Our dataset comprised a total of *n* = 436 LBD, *n* = 53 AD, *n* = 39 PA and *n* = 81 control samples. Sample demographics regarding the percentage of XY males, mean age at death, mean brain weight, percentage of APOE ε4 positive cases, Braak NFT stage, Thal amyloid phase and Lewy body count in the anterior cingulate cortex are summarized in Table 1. Additional sample information are supplied in Supplementary Table 1.

**Table 1:** Overview of sample characteristics

Type	*n* total	XY male	Age, years (mean ± SD)	Brain weight, g, (mean ± SD)	APOE ε4 positive	Thal amyloid phase						Braak NFT stage							Cingulate LB count (mean ± SD)
						0	1	2	3	4	5	0	I	II	III	IV	V	VI	
Control	81	62%	76.1 ± 11.2	1176.4 ± 170.7	12.3%	74	7	–	–	–	–	11	12	26	32	–	–	–	–
PA	39	59%	83.0 ± 6.0	1148.5 ± 116.8	48.7%	–	–	5	25	6	3	1	2	16	20	–	–	–	–
AD	53	55*%*	77.0 ± 9.8	1033.6 ± 118.6	58.5%	–	–	–	4	5	44	–	–	–	–	–	15	38	–
LBD	436	67%	77.1 ± 7.6	1197.1 ± 139.9	47.9%	56	41	39	148	55	97	15	19	116	106	160	12	8	13.2 ± 7.1
LBD (ATS)	178	62%	78.5 ± 7.4	1170.1 ± 145.4	59.0%	–	–	5	68	30	75	–	–	–	–	158	12	8	15.9 ± 7.4
LBD (AS)	161	66%	77.5 ± 6.6	1196.4 ± 131.6	49.7%	–	–	34	80	25	22	2	8	61	90	–	–	–	13.5 ± 6.3
LBD (TS)	2	100%	79.5 ± 3.5	1160.0 ± 84.9	0.0%	2	–	–	–	–	–	–	–	–	–	2	–	–	9.0 ± 1.4
LBD (S)	95	78%	73.7 ± 8.6	1249.7 ± 130.6	25.3%	54	41	–	–	–	–	13	11	55	16	–	–	–	7.7 ± 4.1

Samples used in this study include anterior cingulate cortex tissue from controls (*n* = 81), pathological amyloid (PA, *n* = 39), Alzheimer's disease (AD, *n* = 53) and Lewy body disease (LBD, *n* = 436). LBD cases were additionally stratified into distinct cohorts based on neuropathological criteria: (i) LBD (ATS) = Thal amyloid phase ≥2, Braak neurofibrillary tangle (NFT) stage > III (*n* = 178); (ii) LBD (AS) = Thal amyloid score ≥2, Braak NFT stage ≤III (*n* = 161); (iii) LBD (TS) = Braak NFT score >III, Thal amyloid phase <2 (*n* = 2); and (iv) LBD (S) = Primarily LB pathology, Thal amyloid phase <2, Braak NFT stage ≤III (*n* = 95).

Within the *n* = 436 LBD samples, *n* = 2 cases were classified as BLBD, *n* = 95 cases as TLBD and *n* = 339 cases as DLBD. Specifically, BLBD has no cortical LBs and may have a few in the amygdala, TLBD has no or minimal LBs in multimodal association cortices (midfrontal, superior temporal and inferior parietal), but some LBs in limbic cortices (cingulate gyrus and parahippocampal gyrus). DLBD has LBs in multimodal association cortices (midfrontal, superior temporal and inferior parietal). The LBD cases were additionally stratified into distinct cohorts based on neuropathological criteria. Specifically, cases were categorized into groups exhibiting primarily LB pathology with Thal amyloid phase less than 2 and Braak NFT stage less than or equal to III [referred to as LBD (‘S’); *n* = 95]. Cases with LB pathology coupled with a high Thal amyloid phase equal to or greater than 2 and Braak NFT stage greater than III are referred to as LBD (‘ATS’); *n* = 178. Cases with LB pathology accompanied by a high Thal amyloid score greater than or equal to 2 but with a low Braak NFT stage equal to or less than III are referred to as LBD (‘AS’); *n* = 161. Only two LBD cases exhibited a high Braak NFT score of greater than III but a low Thal amyloid phase of less than 2, referred to as LBD (‘TS’); *n* = 2 (Supplementary Table 1).

### Neuropathological assessment

A single experienced neuropathologist performed systematic and standardized neuropathologic evaluation using formalin-fixed brains. The following brain regions were sampled for histopathologic assessment: six regions of the neocortex, two levels of the hippocampus, a basal forebrain section that includes the amygdala, lentiform nucleus and hypothalamus, anterior corpus striatum, thalamus at the level of the subthalamic nucleus, midbrain, pons, medulla and two sections of the cerebellum, one including the deep nuclei. Paraffin-embedded 5-μm thick sections mounted on glass slides were stained with haematoxylin and eosin (H&E) for general histologic examination and with thioflavin S (Sigma-Aldrich) for assessment of amyloid plaques and NFT. The use of only thioflavin is insufficient for diagnosing argyrophilic grain disease, which is a limitation of this approach. Braak NFT stage and Thal amyloid phase were assigned using thioflavin S fluorescence microscopy, following published criteria.^19-22^ This method, validated in our previous studies,^23^ offers a reliable alternative to tau immunohistochemical staining, particularly in the context of large-scale studies, aligning with National Institute on Aging-Alzheimer's Association guidelines for neuropathological assessment.^22^

To establish a neuropathological diagnosis of LBD,^3^ immunohistochemical staining with the anti-α-synuclein antibody (NACP; rabbit polyclonal; 1:3000; formic acid pretreatment) on sections of the anterior cingulate cortex, hippocampus, basal forebrain and brainstem was performed. LBs were manually counted in the middle frontal (MF), superior temporal (ST), inferior parietal (IP), cingulate (CG) and parahippocampal (PH) gyrus in the field of highest density at ×200 magnification. LBD subtype was classified as brainstem, transitional, or diffuse as described by McKeith *et al*.^3,12^ The tyrosine hydroxylase immunoreactivity (TH-ir) in the dorsolateral and ventromedial putamen was assessed by digital image analysis using immunostained slides of TH (rabbit polyclonal, 1:600; Affinity Bioreagents); a lower TH-ir value corresponds to a greater degree of putaminal dopaminergic degeneration. The neuronal loss in the ventrolateral part of the (SN) was semi-quantitatively scored on H&E-stained sections as follows: 0 = none; 0.5 = minimal; 1 = mild; 1.5 = mild-to-moderate; 2 = moderate; 2.5 = moderate-to-severe; 3 = severe.

All immunohistochemical staining was performed using IHC Autostainer 480S (Thermo Fisher Scientific) with DAKO EnVision™+ reagents and 3,3′-diaminobenzidine (Dako) as the chromogen. Immunostained slides were counterstained with hematoxylin and cover slipped. Unfixed anterior cingulate cortex tissue samples were stored at −80°C until further processing.

### RNA extraction and sequencing alignment

Total RNA was extracted from the anterior cingulate cortex tissue using RNeasy Plus from Qiagen kit following the manufacturer's instructions. RNA quality and integrity were assessed using the RNA integrity number (RIN) measured on an Agilent Bioanalyzer; samples had a mean RIN value of 5.8 (Supplementary Table 1). RNA sequencing (RNAseq) libraries were constructed using Illumina TruSeq Stranded mRNA Library Prep kit V2.

Samples were sequenced to ∼50 million (M) 2 × 100 bp paired-end. Samples were checked for quality using FastQC and aggregated using MultiQC.^24,25^ Data were trimmed to remove adapters using bbduk as part of the bbmap package.^26^ Following trimming, technical lane replicates were merged and paired RNAseq reads were aligned to the Gencode GRCh38.p13 human reference genome informed on the sex chromosome complement of the sample using STAR.^27^ The genetic sex of the sample was first checked by investigating the expression of five Y-linked (*EIF1AY*, *KDM5D*, *UTY*, *DDX3Y*, *RPS4Y1*) genes and one X-linked gene (*XIST*). A sample with a Y chromosome shows expression for most or all Y-linked genes, while samples with at least two X chromosomes show expression for *XIST* (Supplementary Fig. 1).^28^ Samples without evidence of a Y chromosome were aligned to a reference genome with the entire Y chromosome masked with Ns to avoid mismapping of homologous X–Y sequence reads.^28^ Samples with evidence of a Y chromosome were aligned to a reference genome with the Y chromosome pseudoautosomal regions (PARs) masked as those regions are replicated 100% on the X chromosome PARs in GRCh38.p13.^28^

Quantification was performed during the STAR alignment following the twopassMode.^27^ During the alignment, the quantMode flag was employed to obtain gene counts for each gene id.^27^ Alignment metrics regarding the total and fraction of nucleotides within untranslated regions (UTRs), introns, intergenic sequences, peptide-coding sequences (exons) and the number of bases that pass quality filters were obtained following Picard's collectRnaSeqMetrics flag.^29^

### Filtering and normalization

Raw counts were normalized to adjust for sample library size differences using the edgeR CPM (counts per million) function.^30^ Counts were filtered to remove mitochondrial genes and keep only expressed protein-coding genes. A gene is expressed if at least 70% of the smallest group size has a minimum CPM count of 1. Before filtering, there were 60 649 genes, and post-filtering retained 15 208 expressed protein-coding genes. Following filtering, the trimmed mean of values method was used to convert raw library sizes into effective ones.^31^ Sample weights were estimated by the dream function voomWithDreamWeights, which estimates the mean–variance relationship and uses this to compute appropriate observation-level weights.^32^

### Assessing sources of variation

VariancePartition was used to characterize sources of variation inherent within expression data by fitting a linear mixed model among all genes and all samples.^33^ Our exploration encompassed various factors and covariates, including disease type, sex, batch, the proportion of reads aligning to coding, intronic and intergenic regions, RIN and *ENO2* expression as a neuronal biomarker to account for any potential neuronal cell loss due to disease status (Supplementary Fig. 2).^34^

To determine a parsimonious model for differential expression, we applied a Bayesian Information Criterion (BIC)-based approach using a forward stepwise assessment of all variables. The BIC approach applies a penalty term for each added parameter in the model to avoid overfitting. The model with the lowest BIC score included batch, sex, RIN, *ENO2* expression and the percentage of reads aligning to coding, intronic and intergenic regions. We further explored potential sources of variation, including *APOE* ε4+ status, brain weight and age of death. Nonetheless, these factors demonstrated minimal explanatory power concerning the variability within the expression data. Subsequently, our final BIC model was formulated, encompassing ∼0 + disease type + *ENO2* expression + batch + sex + RIN + % coding + % intronic + % intergenic.

### Differential expression analysis

A linear model was fitted to identify differentially expressed genes (DEGs). The modelling was conducted by utilizing the limma lmfit function, which fits an individual model to the expression values of each gene.^35^ Differential expression analyses were performed on each sex, using only female (XX) or male (XY) samples, to identify sex-specific gene dysregulation. We identified significant expression differences using a Benjamini-Hochberg-adjusted *P*-value threshold below 0.05 (5%), referred to as the *q*-value, and we required an absolute log_2_ fold-change greater than 0.25 to ensure the practical relevance of identified differential expression patterns.

### Weighted gene correlation network analysis

To identify co-expression gene modules associated with each disease type, we implemented a weighted gene correlation network analysis (WGCNA).^36^ WGCNA reads stabilized counts as input; we, therefore, input the voom transformed counts described in the ‘Filtering and normalization’ section. We additionally applied the goodSamplesGenes function to determine if any genes or samples should be removed; all genes and samples passed. Hierarchical clustering analysis with the default method ‘complete’ for linking similar gene clusters was implemented. Soft-thresholding powers 1–20 were tested to determine a power that gives a scale-free topology. Here a soft threshold power of 12 was selected to achieve ∼80% signed R^2^ of the model fit (Supplementary Fig. 3). Topological overlap matrices were constructed to find modular structures within the co-expression network using a signed network. Hierarchical clustering was executed to identify interconnected gene clusters representing co-expression modules, resulting in a gene cluster dendrogram by the Dynamic Hybrid tree cut function. Gene modules were then trimmed of genes whose correlation with module eigengene (ME) was less than 0.25. A ME denotes the first principal components of each module. These MEs serve as expression representatives for all genes within a given module. A Pearson's correlation coefficient was then determined for the association between gene modules and clinical variables such as pathology scores, disease type, sex, brain weight, APOE ε4 positive count and age at death, followed by conservative Benjamini & Hochberg multiple test correction. Furthermore, to refine our association between disease and these gene modules, we adopted a strategy to identify DEGs within each module. This approach, denoted as WGNCA + DEGs, was previously outlined,^37^ and facilitated a more granular exploration of disease relevance within the context of co-expression modules.

### Comparison to previously reported Alzheimer's disease RNA sequencing data

To compare our reported results to previously published studies, we accessed gene differential expression data from the Synapse Harmonization Study within the ROSMAP (Religious Orders Study and Rush Memory and Aging Project) dataset,^38^ profiled from the posterior cingulate cortex (PCC) brain region, *n* = 1168. The Synapse Harmonization Study is an extension of the rnaSeqReprocessing study that applied the same tools and methods to all three primary AMP-AD cohort studies: ROSMAP, MSBB (Mount Sinai Brain Bank) and MayoRNAseq. The ROSMAP gene expression changes within the PCC brain region were processed using a similar set of tools and workflow as described for the data presented here, with slight differences. See https://doi.org/10.7303/syn21241740 for details on the rnaSeqReprocessing Synapse Harmonization Study data processing. We compared the log_2_ fold-change (AD/Control) values from the ROSMAP PCC brain region dataset with the log_2_ fold-change (AD/Control) values generated within our anterior cingulate cortex (ACC) brain region cohort to provide a broader perspective on the gene dysregulation within AD.

### Gene set enrichment analysis

To glean comprehensive insights into the biological context and functional significance of differentially expressed genes and correlated gene set modules, we used Metascape.org^39^ and gProfiler^40^ for functional enrichment analysis. Metascape.org offers an integrated platform that amalgamates data from multiple sources, enabling the exploration of gene sets in diverse biological contexts.^39^ Metascape summarizes enriched pathways and allows comparing among gene lists to identify common and uniquely enriched pathways. g:Profiler offers adjusted *P*-values to mitigate false positives due to multiple tests and takes a sorted rank-ordered gene list by log_2_ fold-change as input.^40^

### Consent statement

All human subjects provided consent.

## Results

### Characteristics of each disease group

In this study, gene expression differences were investigated across distinct disease types, namely control, pathological amyloid (predominant amyloid only pathology, PA), AD and LBD (Table 1). The distribution of sex among the disease types was similar, with a higher representation of genetic male (XY) samples overall due to efforts to broadly age and sex match among disease types (Table 1). The higher proportion of male samples in this study can be attributed to tissue availability from the Mayo Clinic brain bank. The distribution of APOE ε4 positive samples were 12.3% for control, 48.7% for PA, 58.5% for AD and 47.9% for LBD (Table 1). Brain weights significantly varied between disease types [H(3) = 56.8, *P*-value <0.001] (Supplementary Fig. 4). Pairwise comparisons using Dunn's test with Bonferroni correction indicated that AD brain weights were significantly different from those of control (*q* < 0.001), PA (*q* < 0.003) and LBD (*q* < 0.001). None of the other pairwise comparisons showed a significant difference. When the LBD cases were further stratified into distinct cohorts based on neuropathological criteria (ATS, AS, TS and S), we again observed significant differences in brain weight among the disease types [H(6) = 75.7, *P*-value <0.001] with AD brains weights significantly different from control, PA and each of the LBD cohorts (*q* < 0.05) (Supplementary Fig. 4). Significant differences in brain weights were additionally observed between LBD (S) and LBD (ATS) cases (*q* = 0.0003), potentially influenced by the degeneration of nigro-striatal circuitry and cortical atrophy associated with the greater neuropathological burden in the LBD (ATS) cases; however, the LBD (ATS) brain weights did not differ significantly from that of the control brains.

### Upregulated inflammatory and downregulated metabolic pathways are enriched in Lewy body disease

**Figure 1:**
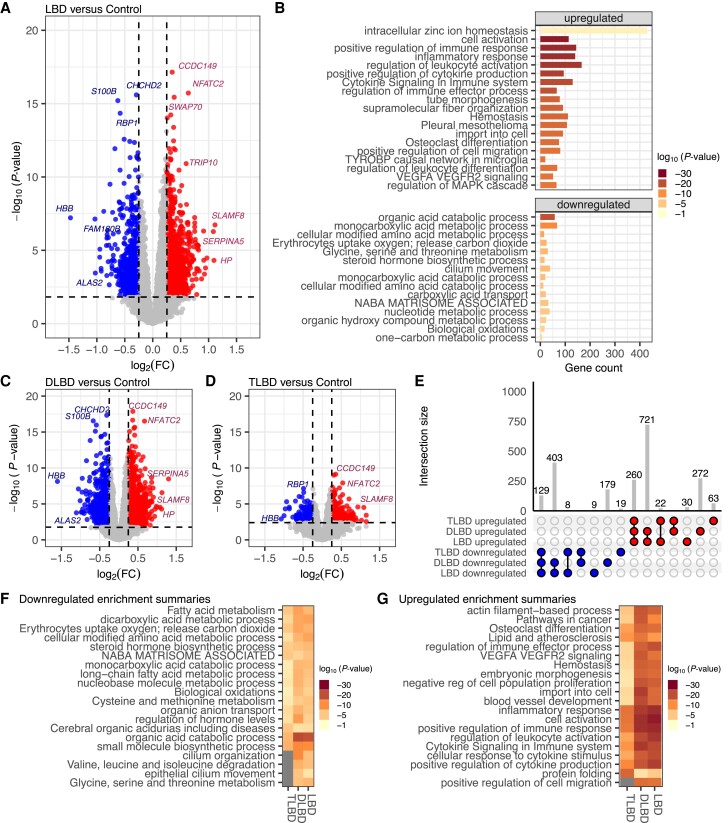
**Upregulation of immune response pathways observed in Lewy body disease versus control cases.** (**A**) Volcano plot of differentially expressed genes for Lewy body disease (LBD, *n* = 436) versus controls (*n* = 81). Genes are differentially expressed, with a false discovery rate (*q-*value) <0.05, downregulated [log_2_ fold-change (log_2_FC) <−0.25] and upregulated (log_2_FC >0.25). Genes not differentially expressed *q-*value <0.05 or absolute log_2_FC <0.25. (**B**) Gene ontology (GO) analysis (metascape.org) showed an upregulation of positive immune response pathways and downregulation of metabolic processes. The *x*-axis shows the gene count contributing to the enrichment pathways listed on the *y*-axis. The colour of the bar indicates the −log_10_*P*-value. Much of the signal observed in **A** is largely owed to the diffuse LBD (DLBD) cases, as seen in (**C**) DLBD (*n* = 339) versus controls (*n* = 81). (**D**) Some transcriptional alterations are observed in transitional LBD (TLBD; *n* = 95) versus controls (*n* = 81). (**E**) The UpSet plot shows the number of shared or unique differentially expressed genes between TLBD, DLBD and LBD. Metascape multi-gene list analysis for (**F**) downregulated and (**G**) upregulated GO terms among TLBD, DLBD, and LBD.

We performed RNAseq from the anterior cingulate cortex in a large series of LBD cases (*n* = 436) and compared them to control samples (*n* = 81). Using a false discovery rate (FDR) *q-*value <0.05 and an absolute log_2_ fold-change >0.25, we identified a total of 1582 DEGs (Fig. 1A and Supplementary Table 2). Of these DEGs, 1033 (65.3%) were upregulated, while 549 (34.7%) were downregulated. We used gene ontology (GO) pathway analysis to understand these transcriptional changes further. The strongest signal from the upregulated genes was from pathways broadly related to inflammation (Fig. 1B and Supplementary Table 3). These pathways included cell activation, inflammatory response, and cytokine production and signalling (Fig. 1B). Upregulated DEGs in these pathways included previously identified microglial activation or ‘disease-associated microglia’ (DAM) genes,^41-43^ including *SPP1*, *CSF1*, *TYROBP* and *TREM2*, as well as several immune-related genes, including *CXCL1*, *CXCL8*, *CCL2* and *CCR5* (Supplementary Table 3). Other enriched pathways included positive regulation of immune responses, hemostasis and regulation of MAPK cascade, arising from upregulated DEGs, including *ANGPT2*, *SERPINA5*, *SERPINA1*, *HSPB1*, *HSPA1A*, *ITGB2*, *NFATC2* and *ICAM1* (Supplementary Fig. 5). Rank-ordered GO analysis with g:Profiler also showed tremendous enrichment of inflammatory pathways for the upregulated DEGs in LBD (Supplementary Fig. 6). GO and pathway analysis of downregulated DEGs showed strong enrichment for organic acid catabolic processes, including *ALDH1A1*, *ADHFE1*, *CARNS1*, *FOLH1*, *ENOSF1* and *GLUD1* (Fig. 1B and Supplementary Fig. 5). There was also a strong downregulation of DEGs related to steroid hormone biosynthetic processes, including *CRH*, *ALB* and *HSD17B8* (Supplementary Fig. 5). Rank order analysis with g:Profiler similarly showed downregulated DEGs strongly enriched in metabolic and steroid hormone dysfunction pathways (Supplementary Fig. 6). In summary, the LBD cases had a significant upregulation of inflammatory pathways and downregulation of metabolic pathways.

### Robust transcriptional alterations in Lewy body disease are attributed to diffuse Lewy body disease

We next sought to determine how much of the transcriptional dysregulation in the LBD cases was attributed to diffuse (DLBD) versus transitional (TLBD) subtypes. A direct comparison of the DLBD cases (*n* = 339) versus controls (*n* = 81) identified 1253 upregulated and 711 downregulated genes (Fig. 1C). Within the TLBD cases (*n* = 95) versus controls, we only observed 345 upregulated and 156 downregulated DEGs (Fig. 1D). Considerable overlap was observed between the genes showing significant differences in the larger LBD cohort compared to those identified in the DLBD subtype as shown in the UpSet plot (Fig. 1E).

Comparative GO analysis identified pathways commonly or uniquely enriched in downregulated (Fig. 1F) and upregulated (Fig. 1G) DEGs between TLBD, DLBD and the overall LBD cohort. Several catabolic and metabolic pathways were significantly downregulated in TLBD, DLBD and LBD cases, while some processes were not significantly downregulated in TLBD (Fig. 1F and Supplementary Table 4). In the analysis of upregulated DEGs, protein folding emerged as a more significantly enriched pathway in TLBD cases, compared with DLBD and LBD subtypes (Fig. 1G and Supplementary Table 4). In general, DLBD exhibited more significantly enriched pathways of upregulated and downregulated GO terms than TLBD or the total LBD cohort, highlighting a potentially higher degree of dysregulation in the DLBD subset.

DLBD versus TLBD showed marked differences, with unique gene expression signatures identified between these distinct LBD subtypes. Specifically, 74 genes were upregulated, while 49 genes were downregulated in DLBD compared to TLBD (Supplementary Fig. 7 and Supplementary Table 2). GO analysis of the upregulated genes in DLBD compared to TLBD highlighted differences in myelination, neurodevelopmental processes and cellular maintenance (Supplementary Fig. 7). Conversely, the downregulated DEGs were enriched in pathways related to endocrine regulation and hormone levels (Supplementary Fig. 7). These findings underscore the differences in DLBD and TLBD, likely due to the pathological burdens between these LBD subtypes.

We next wanted to assess whether the presence of tau pathology contributes significantly to gene dysregulation within LBD. We analysed the larger cohort of LBD cases and the LBD subtypes, DLBD and TLBD, stratified by tau pathology status. Based on Braak *et al*.,^44^ there should be no NFT pathology detected in the anterior cingulate cortex until Braak stage IV. Therefore, in this context, tau positive is defined as a Braak NFT stage equal or greater than IV, while tau negative corresponds to a Braak NFT stage equal or less than III. Remarkably, a comparison between tau positive and tau negative cases, within each LBD subtype as well as the larger LBD cohort, did not reveal significant DEGs (Supplementary Fig. 8). Our findings suggest that while tau is present in a substantial number of LBD cases, it does not substantially influence the gene expression profile within this disease. Likewise, we sought to elucidate the impact of *APOE* ε4 status on gene expression, given its strong genetic association with LBD.^15^ Comparison of DLBD cases carrying an *APOE* ε4 allele (*n* = 188) and those without an *APOE* ε4 allele (*n* = 151), revealed no significant DEGs (Supplementary Fig. 8). Although less *APOE* ε4 allele positive cases in the TLBD cases, comparison of TLBD cases carrying an *APOE* ε4 allele (*n* = 19) and those without an *APOE* ε4 allele (*n* = 76), also revealed no significant DEGs (Supplementary Fig. 8). This suggests that *APOE* ε4 status on gene expression is minimal in the later stages of DLBD or during the intermediate stage of TLBD. We also explored the influence of concomitant TDP-43 pathology in our LBD cases, given its presence in many neurodegenerative diseases.^45^ Among the *n* = 134 TDP-43 positive (TDP-43+) and *n* = 289 TDP-43 negative (TDP-43−) cases analysed (Supplementary Table 1), no significant DEGs were detected (Supplementary Fig. 8). This suggests that TDP-43 pathology may not substantially influence gene expression alterations in the anterior cingulate cortex within LBD cases. At this level of bulk tissue analysis, our data indicate that tau, APOE genotype and TDP-43 pathology do not drive substantial gene expression alterations in LBD despite the fact that our data should have sufficient sample sizes to detect these.

### Lewy body disease cases show upregulation of cytokine signalling, regardless of co-pathology

**Figure 2:**
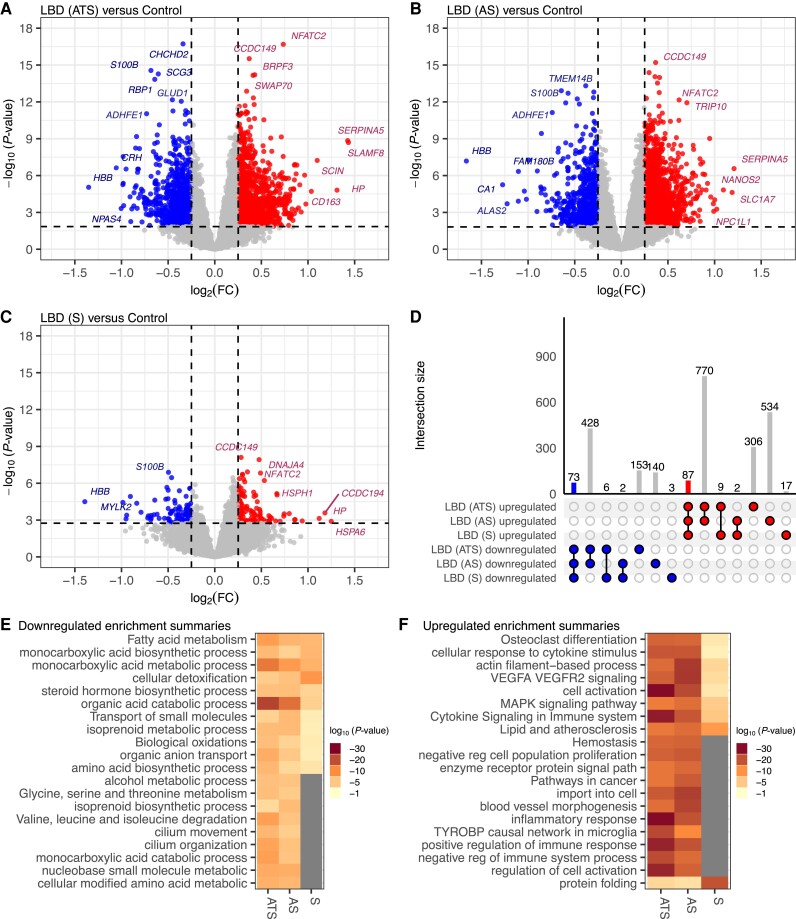
**Upregulation of cytokine signalling and downregulation of fatty acid metabolism among Lewy body disease cases, irrespective of co-pathology**. Volcano plot of differentially expressed genes in cases with Lewy body disease (LBD). (**A**) Thal amyloid phase ≥ 2 and Braak neurofibrillary tangle (NFT) stage > III [LBD (ATS), *n* = 178], (**B**) Thal amyloid phase ≥ 2 and Braak NFT stage ≤ III [LBD (AS), *n* = 161] and (**C**) Thal amyloid phase <2 and Braak NFT stage ≤ III [LBD (S), *n* = 95] versus controls (*n* = 81). Genes are differentially expressed, with a false discovery rate (*q-*value) < 0.05, downregulated [log_2_ fold-change (log_2_FC) < −0.25] and upregulated (log_2_FC >0.25). Genes not differentially expressed *q-*value <0.05 or absolute log_2_FC <0.25. (**D**) The UpSet plot shows the number of shared or unique differentially expressed genes between LBD (ATS), LBD (AS) and LBD (S). Metascape multi-gene list analysis for (**E**) downregulated and (**F**) upregulated gene ontology terms among LBD (ATS), LBD (AS) and LBD (S) show downregulation of fatty acid metabolism and upregulation of protein folding and cytokine signalling in the immune system.

The LBD cases were categorized into distinct groups based on neuropathological criteria to assess the impact of amyloid-β plaque and NFT co-pathology on transcriptional dysregulation. LBD groups included: LBD (ATS) with LB pathology, Thal amyloid phase ≥2 and Braak NFT stage > III (*n* = 178); LBD (AS) with LB pathology, Thal amyloid score ≥2 and Braak NFT stage ≤ III (*n* = 161); and LBD (S) with primarily LB pathology, Thal amyloid phase <2 and Braak NFT stage ≤ III (*n* = 95) (Supplementary Table 1). Each LBD group was compared to controls to identify uniquely or commonly dysregulated genes. A direct comparison of LBD cases with amyloid and tau and synuclein pathology (ATS) versus controls revealed 1172 upregulated and 660 downregulated genes (Fig. 2A and Supplementary Table 2). Similarly, LBD cases with just amyloid and synuclein pathology (AS) versus controls exhibited 1393 upregulated and 643 downregulated DEGs (Fig. 2B). In contrast, LBD cases with just synuclein pathology (S) versus controls showed fewer DEGs, with only 115 upregulated and 84 downregulated genes (Fig. 2C). Despite the smaller number of DEGs in LBD (S) versus controls, there was a set of DEGs that overlap among all LBD groups compared to controls (Fig. 2D), indicating a shared dysregulated gene response that might be attributed to LB pathology.

We conducted multi-gene list enrichment analysis to delve deeper into the functional implications of the dysregulated gene expression observed in the LBD cases. This comparative GO analysis identified pathways commonly or uniquely enriched in downregulated (Fig. 2E) and upregulated (Fig. 2F) DEGs across LBD (ATS), LBD (AS) and LBD (S) cohorts compared to controls (Supplementary Table 4). Several metabolic processes exhibited significant downregulation in all LBD cases, though a few were not significantly downregulated in LBD (S) (Fig. 2E). Conversely, in the analysis of upregulated DEGs, protein folding emerged as a more significantly enriched pathway in LBD (S) cases compared to LBD (ATS) and LBD (AS) (Fig. 2F), an observation that was similarly observed when comparing among TLBD and DLBD relative to controls (Fig. 1G). These observations may suggest that genes involved in protein folding are more upregulated during earlier stages of LBD. Generally, LBD (ATS) and LBD (AS) demonstrated more significantly enriched pathways of upregulated and downregulated GO terms compared to LBD (S), indicating a potentially higher degree of dysregulation in the presence of co-pathology (Fig. 2E and F).

To gain deeper insights into the shared dysregulation among the neuropathologically defined LBD cohorts, we conducted a GO analysis focusing exclusively on the commonly downregulated and upregulated DEGs. Among the 73 commonly downregulated genes, pathways associated with cellular detoxification and metabolic processes were significantly enriched (Supplementary Fig. 9). Conversely, the 87 commonly upregulated genes exhibited enrichment in pathways related to protein folding, circadian regulation and innate immune response (Supplementary Fig. 9). A substantial portion of the DEGs observed in LBD (ATS) and LBD (AS) did not overlap with those identified in the LBD (S) versus control comparison (Fig. 2D). To address these discrepancies, we compared the log_2_ fold-change values of LBD (ATS)/control and LBD (AS)/control with those of LBD (S)/control to ascertain whether the uniquely differentially expressed genes in each cohort displayed consistent expression trends (Supplementary Fig. 9). Our analysis revealed that most genes across these comparisons exhibited concordant directionality in their fold-change. Pearson's correlation coefficient of log_2_ fold-change LBD (AS)/control versus LBD (ATS)/control for all genes (q-value ≤1) was *r* = 0.92 with a *P*-value <2.2 × 10^−16^, indicating a robust correlation. The Pearson's correlation coefficient for all genes (*q*-value ≤1) of log_2_ fold-change LBD (S)/control versus LBD (AS)/control and LBD (ATS)/control was *r* = 0.75 with a *P*-value <2.2 × 10^−16^ and *r* = 0.81 with a *P*-value <2.2 × 10^−16^, respectively, indicating strong correlations. In the comparisons of the overall LBD, LBD (ATS) and LBD (AS) versus control, we observed upregulation of activation/DAM genes,^41-43^ such as *SPP1*, *CSF1*, *TYROBP* and *TREM2*. These genes were not identified as significantly (i.e. *q*-value <0.05) differentially expressed in the LBD (S) versus control analysis, although they were found to be upregulated upon further investigation (Supplementary Fig. 9). In summary, although fewer DEGs were identified in the LBD (S) versus control comparison relative to the LBD (ATS) and LBD (AS) versus control analyses, the directional trends of the genes in LBD (S) versus control were similar, suggesting there may be a ‘dose-effect’ of pathology on gene expression.

### Alzheimer's disease cases display upregulation of inflammatory and down-regulation of metabolic pathways

**Figure 3:**
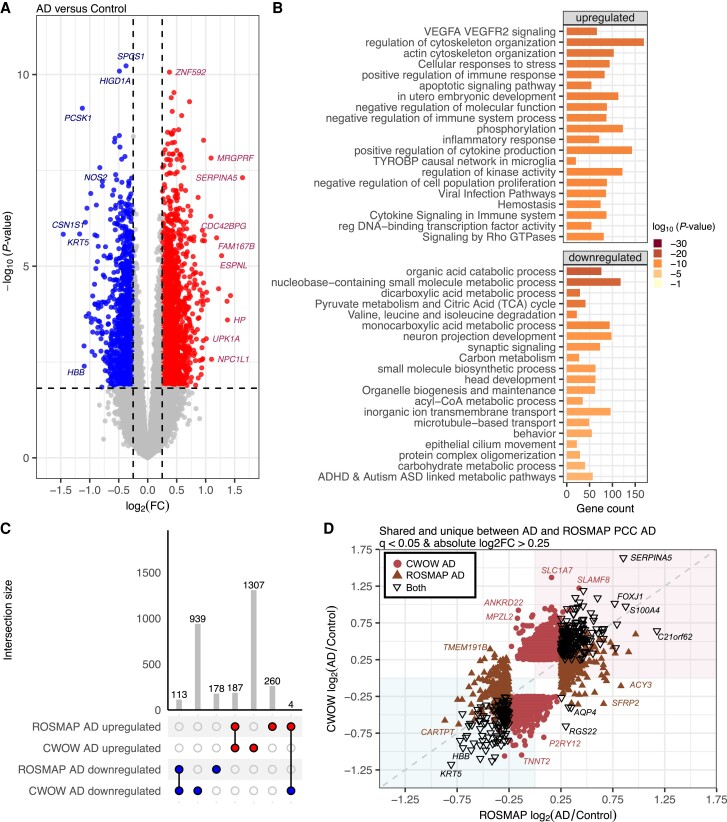
**Gene differential expression between Alzheimer's disease and controls were enriched in cellular response to stress and *TYROBP* causal network in microglia**. (**A**) Gene differential expression between Alzheimer's disease (AD, *n* = 53) and controls (*n* = 81). (**B**) Upregulated genes are enriched in vasculature, immune and cell death. Downregulated genes are enriched in metabolic and synaptic related pathways. (**C**) UpSet plot showing the number of differentially expressed genes [*q-*value <0.05 and absolute log_2_ fold-change (log_2_FC) > 0.25] shared or unique between AD versus control reported in this Center without Walls (CWOW) study and the previously reported Religious Orders Study and Rush Memory and Aging Project (ROSMAP) Synapse Harmonization Study. (**D**) A scatter plot of the log_2_ fold-change of ROSMAP AD versus control (*x*-axis) and CWOW AD versus control (*y*-axis) reveals differentially expressed genes common to both datasets (black open triangles), unique to the CWOW dataset (maroon filled circles) or unique to the ROSMAP dataset (brown filled triangles). Genes within the teal shaded area (*bottom left*) had a negative log_2_FC, and genes within the pink shaded area (*top right*) had a positive log_2_FC in both datasets.

We next analysed gene expression patterns in our AD cases. The comparison of AD samples (*n* = 53) with controls (*n* = 81) identified 2704 DEGs using the same strict *q*-value <0.05 and absolute log_2_ fold-change >0.25 thresholds (Fig. 3A and Supplementary Table 2). Among these DEGs, 1597 (59.1%) were upregulated, while 1107 (40.9%) were downregulated. A GO analysis of the 1597 significantly upregulated genes pointed towards processes related to vascular integrity, immune responses, cellular stress and structural dynamics (Fig. 3B and Supplementary Table 3). DEGs in these pathways included heat shock proteins (*HSPB1* and *HSPA1A*) and several activation/DAM genes (*CSF1*, *TYROBP* and *TREM2*)^41-43^ (Supplementary Fig. 10). Rank order analysis in g:Profiler showed similar strong upregulated enrichment in immune responses and cellular stress (Supplementary Fig. 11). A GO analysis of the 1107 downregulated genes showed enrichment for pathways involved in energy metabolism, neurotransmission, cellular structure and developmental processes (Fig. 3B). DEGs with the strongest downregulation in the organic acid catabolic process pathway included: *TYRP1*, *ALDH1A1*, *NOS2*, *TDO2* and *DCT* (Supplementary Fig. 10). There was also a strong downregulation of DEGs related to behaviour, including *CRH*, *SLC12A*, *TPBG*, *TAC1* and *EGR1*, and acyl-CoA metabolic process, including *TYRP1*, *TDO2*, *ACSL6*, *AADAT*, *SUCLG2* and *HSD17B8* (Supplementary Fig. 10). Rank order analysis in g:Profiler showed similar strong downregulated enrichment in catabolic pathways (Supplementary Fig. 11).

To benchmark and compare our data to previously published datasets, we accessed gene differential expression data of posterior cingulate cortex tissue from the Synapse Harmonization Study within the ROSMAP dataset (*n* = 1168 samples). Though a slightly different brain region, we reasoned that it would have at least some shared changes with our anterior cingulate cortex data as both neocortical areas are impacted by pathology. The DEG analysis in ROSMAP AD compared to control samples reported 246 downregulated genes and 426 upregulated genes meeting the criteria of *q-*value <0.05 and absolute log_2_ fold-change >0.25. We observed 113 commonly downregulated genes between our dataset reported here and the previously reported ROSMAP DEGs (Fig. 3C). Similarly, 187 genes were identified as commonly upregulated between the two datasets. The existence of commonly dysregulated genes suggests a degree of consistency across datasets and brain regions. Four genes show an opposite direction in expression between the two datasets: *AQP4*, *RGS22*, *TMEM220* and *TMEM47*, which are upregulated in ROSMAP but downregulated in our data (Fig. 3C). A considerable portion, 88.1%, of DEGs reported in this study [Lewy body dementia Center without Walls (CWOW)] and ROSMAP datasets did not overlap, suggesting context-dependency of gene expression changes in different cohorts (Fig. 3C). We further investigated these discrepancies by comparing the log_2_ fold-change (AD/control) values from ROSMAP with the log_2_ fold-change (AD/control) values generated in our CWOW study to determine if the uniquely differentially expressed genes in each dataset show the same up or downregulated expression (Fig. 3D). We observed that most DEGs between the two datasets show the same directionality as being up or downregulated in AD compared to controls. Pearson's correlation coefficient of the log_2_ fold-change (AD/Control) for all 2988 DEGs shared and unique between ROSMAP and CWOW was *r* = 0.64 with a *P-*value <2.2 × 10^−16^, indicating a strong correlation. When examining the 304 DEGs common to both datasets (black triangles in Fig. 3D) the correlation is even stronger, *r* = 0.92 and *P*-value <2.2 × 10^−16^. Thus, this analysis identified a set of genes commonly dysregulated in Alzheimer's disease compared to controls in two independent datasets from two independent brain regions.

### Shared and distinct gene dysregulation in Lewy body disease and Alzheimer's disease

**Figure 4:**
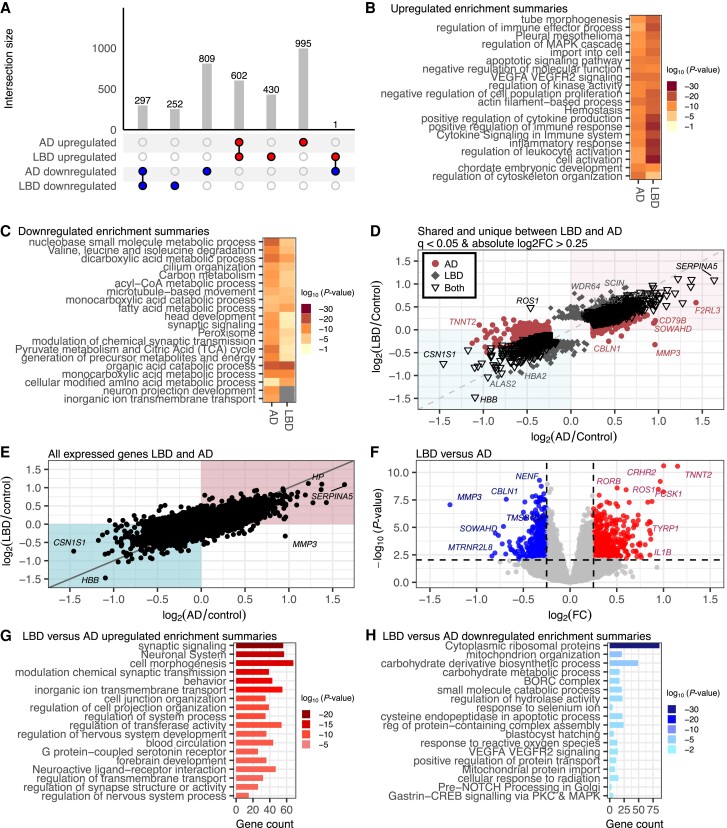
**Shared and unique transcriptional alterations between Lewy body disease and Alzheimer's disease.** (**A**) UpSet plot comparing differentially expressed genes (*q-*value <0.05 and absolute log_2_ fold-change >0.25) found in Lewy body disease (LBD) and Alzheimer's disease (AD) versus control. (**B**) Multi-list gene ontology (GO) analysis of the upregulated differentially expressed genes (DEGs) shows shared enrichment of vasculature, immune and MAPK cascade related pathways. (**C**) Downregulated DEGs are commonly enriched in metabolic pathways. (**D**) A scatter plot of the log_2_ fold-change of AD versus control (*x*-axis) and LBD versus control genes (*y*-axis) reveals DEGs common to both diseases (black open triangles), unique to AD (maroon filled circles) and unique to LBD (grey filled diamonds). Genes within the teal shaded area (*bottom left*) had a negative log_2_ fold-change in both AD and LBD, and genes within the pink shaded area (*top right*) had a positive log_2_ fold-change in both. (**E**) Scatter plot of all expressed genes (*q-*value ≤1) between LBD versus control and AD versus control, regardless of fold-change direction or significance value. (**F**) Volcano plot of genes differentially expressed between LBD (*n* = 436) versus AD (*n* = 53). (**G**) GO analysis of genes upregulated in LBD versus AD are largely enriched in synaptic and neurodevelopmental pathways. (**H**) Genes downregulated in LBD versus AD are enriched in ribosomal, mitochondrial and metabolic pathways. log_2_FC = log_2_ fold-change.

To investigate the presence of a common set of dysregulated genes across different neurodegenerative diseases, we conducted a comparative analysis of DEGs in LBD and AD cases compared to the control cases. As shown in Fig. 1A, the comparison of LBD cases to controls revealed 1582 DEGs (Fig. 1A). In comparing AD cases to controls, we identified 2704 DEGs (Fig. 3A). Comparing these DEG sets, we identified a core set of commonly altered genes, 297 down and 602 upregulated, across both LBD and AD cases (Fig. 4A). This observation suggests that there exists a fundamental set of genes that respond to neuropathological changes across these two distinct neurodegenerative conditions. Only one gene showed an opposite expression pattern, *ROS1* (ROS proto-oncogene 1 receptor tyrosine kinase), that was upregulated in LBD but downregulated in AD (Fig. 4A). A large portion of the DEGs, specifically 73.4%, were not shared between LBD and AD. This disparity in gene expression alterations may point towards disease-specific gene dysregulation within these neurodegenerative conditions.

We performed enrichment analysis with multi-gene list input to gain deeper insights into the functional implications of the dysregulated gene expression observed in LBD and AD (Fig. 4B and Supplementary Table 4). This analysis aimed to elucidate the biological processes and pathways associated with the 1033 and 1597 upregulated genes identified in the LBD and AD cases compared to controls, respectively. We observed a substantial overlap in enriched GO terms between the two conditions, including inflammation, immune response modulation and cellular migration (Fig. 4B). While these pathways were shared between LBD and AD, we observed generally more significant *P*-values for the enrichment of these terms in the LBD group (Fig. 4B), indicating a potentially stronger association between the dysregulated genes and these biological processes in LBD. We additionally assessed the overall shared enrichment of downregulated genes using multi-gene list input (Fig. 4C and Supplementary Table 4). Commonly downregulated pathways involve cellular and metabolic processes that include microtubule dynamics, neurotransmission and metabolic pathways, suggesting disrupted cellular communication. Strikingly, some pathways were uniquely enriched in AD versus control, including neuron projection development and inorganic ion transmembrane transport (Fig. 4C). Largely, the GO enrichment analysis highlights shared functional themes associated with LBD and AD that may be reflective of, or contribute to, neurodegeneration.

To characterize the shared dysregulated genes observed in LBD and AD, we investigated the log_2_ fold-change (AD/Control) compared with the log_2_ fold-change (LBD/Control) values to determine if the unique DEGs in each cohort show the same expression patterns (Fig. 4D). We observed that most DEGs between the two disease types show the same directionality as being up or downregulated compared to controls. Pearson's correlation coefficient of the log_2_ fold-change for all 3386 DEGs shared and unique between LBD and AD was *r* = 0.84, with a *P*-value <2.2 × 10^−16^, indicating a strong correlation. When examining log_2_ fold-change for all genes regardless of significance (*q*-value ≤1), the correlation was still strong, with *r* = 0.73 and *P*-value <2.2 × 10^−16^ (Fig. 4E).

Next, we directly compared LBD (*n* = 436) to AD (*n* = 53) to identify genes that are differentially expressed between these two distinct neurodegenerative conditions (Fig. 4F). In this direct comparison, we observed 899 DEGs, of which 539 were upregulated and 360 were downregulated (Fig. 4F and Supplementary Table 2). Pathway analysis showed that the strongest signal from the upregulated genes was from pathways broadly related to synaptic signalling, behaviour and neuronal system (Fig. 4G and Supplementary Table 3). Upregulated DEGs in the synaptic signalling pathway included *SV2C*, *CARTPT*, *SLC17A6*, *CHRM2* and *HTR2C* (Supplementary Fig. 12). Other upregulated enriched pathways included regulation of transferase activity, including genes *TNNT2*, *MAS1*, *ROS1*, *KNG1* and *IL1B*; as well as forebrain development, including genes *MAS1*, *PCSK1*, *GDF7*, *DLX6*, *KCNC2* and *EMX1* (Supplementary Fig. 12). Rank-order pathway analysis with g:Profiler likewise was enriched in signalling pathways and forebrain development for the upregulated DEGs in LBD versus AD (Supplementary Fig. 13). Pathway analysis of downregulated DEGs showed strong enrichment for cytoplasmic ribosomal proteins, mitochondrion organization and small molecule catabolic process, including numerous ribosomal genes (Supplementary Fig. 12). Rank order analysis in g:Profiler showed similar strong enrichment in ribosome, cytoplasmic and mitochondrion organization pathways downregulated in LBD compared to AD (Supplementary Fig. 13). These findings collectively underscore distinct molecular underpinnings of these two neurodegenerative diseases.

### Lewy body disease exhibit decreased cytoplasmic ribosomal activity compared to Alzheimer's disease

**Figure 5:**
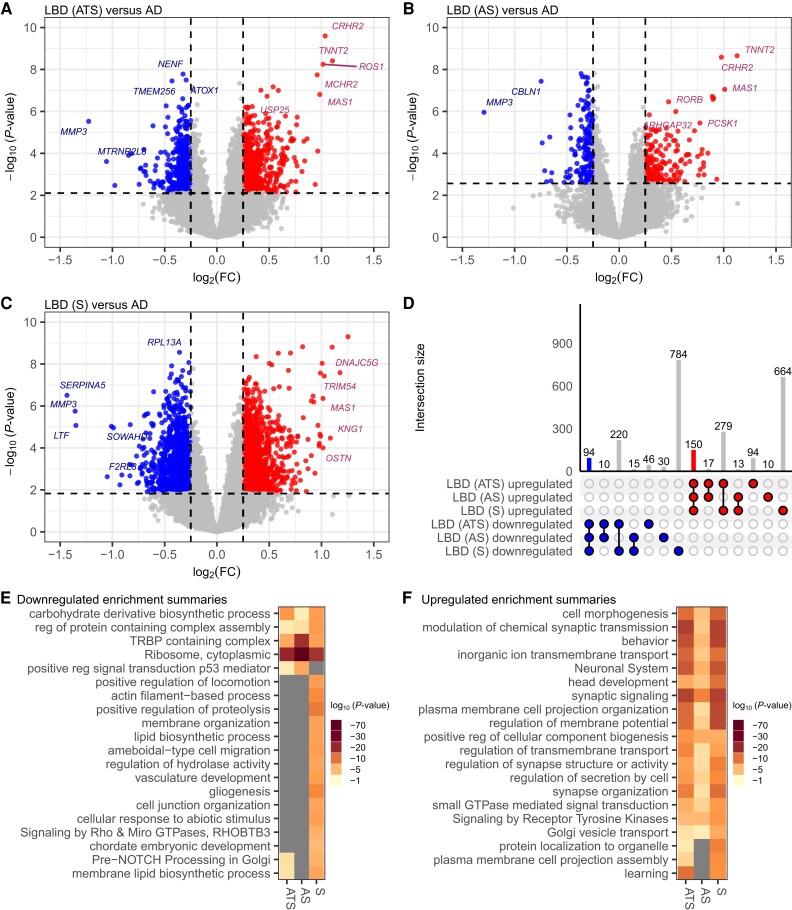
**Decreased cytoplasmic ribosomal activity and upregulated synaptic signalling in Lewy body disease (LBD) versus Alzheimer's disease (AD), regardless of co-pathology**. Volcano plot of differentially expressed genes for (**A**) LBD (ATS) (*n* = 178), (**B**) LBD (AS) (*n* = 161) and (**C**) LBD (S) (*n* = 95) versus Alzheimer's disease (*n* = 53; AD). (**D**) UpSet plot shows the number of shared or unique differentially expressed genes between LBD (ATS), LBD (AS) and LBD (S). Metascape multi-gene list analysis for (**E**) downregulated and (**F**) upregulated gene ontology terms among LBD (ATS), LBD (AS) and LBD (S) show significant downregulation of cytoplasmic ribosome and upregulation of synaptic signalling. LBD (ATS) = Thal amyloid phase ≥2, Braak neurofibrillary tangle (NFT) stage > III; LBD (AS) = Thal amyloid score ≥2, Braak NFT stage ≤III; LBD (TS) = Braak NFT score >III, Thal amyloid phase <2; LBD (S) = Primarily LB pathology, Thal amyloid phase <2, Braak NFT stage ≤II; log_2_FC = log_2_ fold-change.

Stratifying LBD cases by neuropathological criteria, we observed varied degrees of dysregulation compared to AD. LBD with high Thal amyloid and Braak NFT stage (ATS) versus AD displayed 540 upregulated and 370 downregulated genes (Fig. 5A). LBD with high Thal amyloid phase (AS) versus AD showcased 190 upregulated and 149 downregulated differentially expressed genes (Fig. 5B). Notably, LBD without significant co-pathology (S) versus AD exhibited the most profound alterations, with 1106 upregulated and 1113 downregulated genes (Fig. 5C), possibly attributed to LBD (S) having the most distinct pathology to AD compared to LBD (AS) and (ATS). Furthermore, we identified a shared pool of dysregulated genes among the LBD cohorts compared to AD, comprising 94 commonly downregulated and 150 commonly upregulated genes (Fig. 5D). These findings suggest a unique set of genes altered in LB pathology cases irrespective of co-pathology compared to AD.

To gain deeper insights into the functional implications of the dysregulated genes observed in each LBD neuropathologically defined group versus AD, we performed enrichment analysis with multi-gene list input. This analysis aimed to characterize the biological processes and pathways associated with the downregulated and upregulated genes in each LBD neuropathologically defined group versus AD cases. We observed an overlap in enriched GO terms between the LBD groups (ATS, AS, S), including downregulation of genes involved in the cytoplasmic ribosome and upregulation of genes involved in behaviour, neuronal system and synaptic signalling (Fig. 5E and F). While these pathways were shared between LBD (ATS), LBD (AS) and LBD (S), we observed generally more significant *P*-values for the enrichment of these terms in the LBD (S) group (Fig. 5E and F). Furthermore, several downregulated pathways were uniquely enriched in LBD (S) versus AD, including vasculature development, gliogenesis and cell junction organization (Fig. 5E). Largely, the upregulated GO enrichment analysis, highlights shared functional themes among the LBD neuropathology groups (ATS, AS, S) compared to AD which could point to a role for LB pathology, specifically.

### Females show more robust transcriptional alteration compared to males across disease types

**Figure 6:**
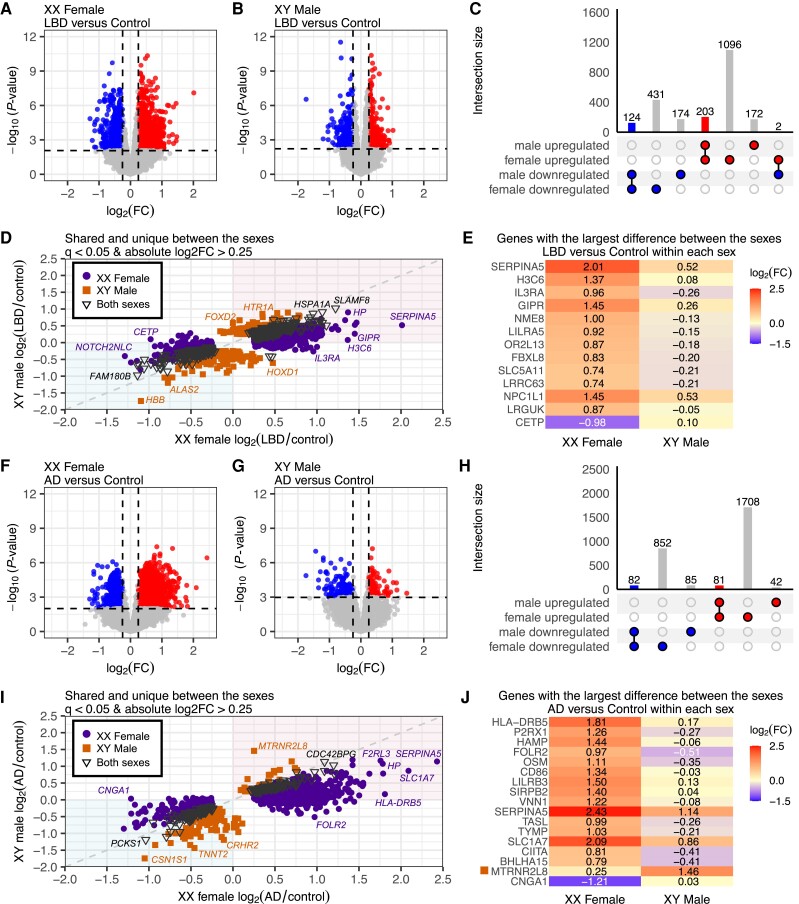
**Sex-specific and sex-shared gene differential expression within disease types.** Volcano plots showing genes differentially expressed between (**A**) XX females with Lewy body disease (LBD; *n* = 142) compared to female controls (*n* = 31) and (**B**) XY males with LBD (*n* = 294) compared to male controls (*n* = 50). (**C**) UpSet plot comparing sex-specific differential expression in LBD [*q* < 0.05; absolute log_2_ fold-change (log_2_FC) > 0.25]. (**D**) Scatter plot of the differentially expressed genes identified in female LBD (*x*-axis) and male LBD cases (*y*-axis). (**E**) A heat map of the genes with the largest difference in expression between the female- and male-only analyses. All genes depicted in **E** are significantly differentially expressed in females. (**F–J**) The above repeated for Alzheimer's disease (AD) versus controls within each sex. All genes shown in **J** are significantly differentially expressed in females, excluding *MTRNR2L8*, which is uniquely significantly differentially expressed in male AD individuals.

The transcriptional alterations within each genetic sex were examined for each pathologic type to identify sex-specific and sex-shared gene differential expression patterns. The comparison of XX female LBD cases (*n* = 142) to XX female controls (*n* = 31) revealed numerous significant alterations with 1856 DEGs (Fig. 6A and Supplementary Table 5). Similarly, XY male LBD cases (*n* = 294) compared to male XY controls (*n* = 50) had 675 DEGs (Fig. 6B and Supplementary Table 5). Strikingly, despite a smaller sample size, we observed a higher number of DEGs in the XX female analysis (Fig. 6A–C). To further characterize the shared and unique dysregulation of genes between the sexes, we investigated the log_2_ fold-change (LBD/control) within XX females compared with the log_2_ fold-change (LBD/control) within XY males to determine if the unique DEGs in each sex show the same up or downregulated expression. We observed that most DEGs between the sexes show the same directionality as being up or downregulated (Fig. 6D). Pearson's correlation coefficient of the log_2_ fold-change for all 2202 DEGs shared and unique between the sexes was *r* = 0.75 and *P*-value <2.2 × 10^−16^, indicating a strong correlation. When examining all genes regardless of significance (*q*-value ≤1), the correlation was still strong, *r* = 0.60 and *P*-value <2.2 × 10^−16^. Interestingly, two genes show a significant opposite expression pattern between the sexes, *GTPBP6* and *PPP2R3B*, which were upregulated in XX females and downregulated in XY males (Fig. 6C and D). These genes are located on the X chromosome pseudoautosomal region, PAR. Overall, XX females showed a greater transcriptional dysregulation than XY males, even for genes with the same direction bias in both sexes (Fig. 6D and E).

Similarly, we conducted sex-specific DEG analyses for AD cases compared to controls (Fig. 6F and G). The comparison of XX female AD cases (*n* = 24) to XX female controls (*n* = 31) revealed numerous significant alterations with 2723 DEGs (Fig. 6F and Supplementary Table 5). The XY male analysis of AD cases (*n* = 29) compared to male XY controls (*n* = 50) revealed only 290 DEGs (Fig. 6G and Supplementary Table 5). Consistent with the LBD findings, we again observed a greater number of DEGs in XX females than in XY males despite the larger sample size of the XY male groups (Fig. 6H). To further quantify the shared and unique gene dysregulation between the sexes, we investigated the log_2_ fold-change (AD/Control) between the sexes (Fig. 6I). We again observed that most DEGs between the sexes show the same directionality as being up or downregulated (Fig. 6I). Pearson's correlation coefficient of the log_2_ fold-change for all 2850 DEGs shared and unique between the sexes was *r* = 0.79 and *P*-value <2.2 × 10^−16^, indicating a strong correlation. When examining all genes (*q*-value ≤1) the correlation was still strong, with *r* = 0.64 and *P*-value <2.2 × 10^−16^. No genes within the AD versus controls showed significant opposite expression between the sexes (Fig. 6H and I), though several genes show a much stronger upregulation within XX females compared to XY males (Fig. 6J). In summary, XX females exhibited more robust transcriptional dysregulation within each disease type compared to XY males.

To understand the effect of sex in gene dysregulation within AD, we compared our data to previously published ROSMAP posterior cingulate cortex tissue from the Synapse Harmonization Study dataset. Within the ROSMAP dataset, 532 DEGs were observed in the XX female and 273 DEGs were observed in the XY male only AD versus control analyses, meeting the criteria of *q-*value <0.05 and absolute log_2_ fold-change >0.25 (Supplementary Fig. 14). As observed in the data reported here, the ROSMAP data also reported more DEGs in the female only analysis, suggesting more robust transcriptional dysregulation in females than males.

### Weighted gene correlation network analysis identifies gene sets associated with neuropathology

**Figure 7:**
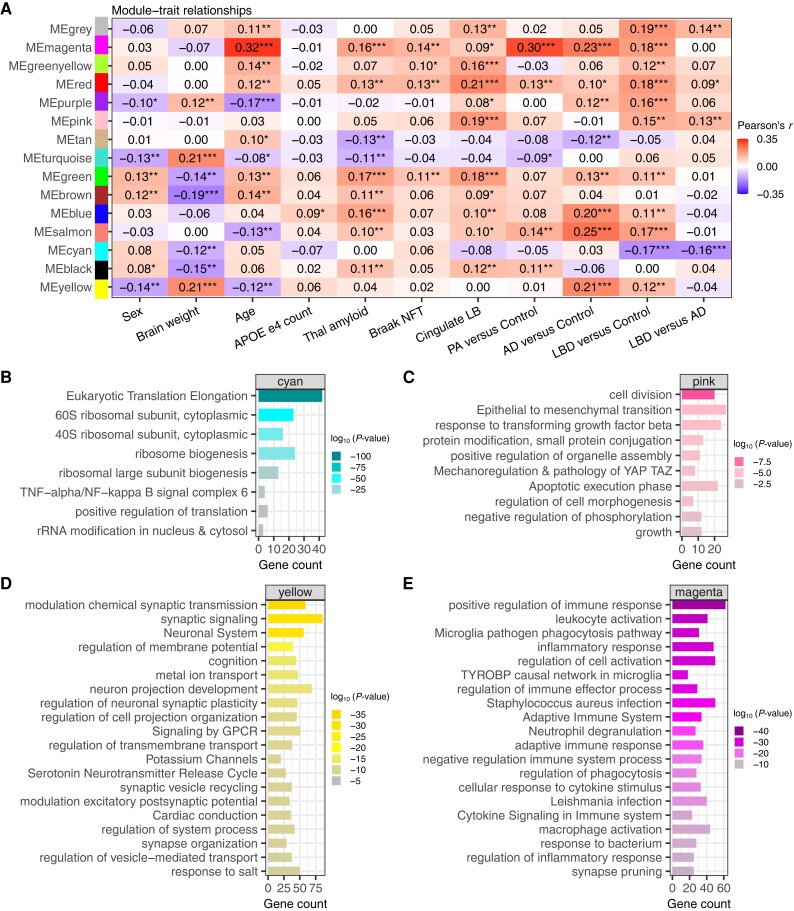
**Gene set modules associated with clinical traits, pathology, and disease type identified by weighted gene correlation network analysis**. (**A**) Pearson correlation coefficient heat map shows the relationships between gene set modules and clinical traits, pathology, disease versus control, and Lewy body disease (LBD) versus Alzheimer's disease (AD). *Significant association between the gene modules (Benjamini-Hochberg multiple test correction; *q < 0.05, **q < 0.01, ***q < 0.001). Gene ontology analysis of modules in **B**, cyan; **C**, pink; **D**, yellow; and **E**, magenta. NFT = neurofibrillary tangle.

We conducted a Weighted Gene Correlation Network Analysis (WGCNA) to identify gene sets associated with each disease type. Although our pathological amyloid (PA) cases only have three DEGs that met our significance criteria, we observed a handful of DEGs in the XX female only analysis (Supplementary Fig. 15 and Supplementary Tables 2 and 5); thus, we included PA data along with LBD, AD and controls. Our analysis identified 15 modules (Supplementary Fig. 16), each representing a set of at least 50 co-expressed genes (Supplementary Fig. 16 and Supplementary Table 6). Pearson's correlation between gene modules and disease status identified several modules that were significantly associated with neurodegeneration (Fig. 7A). Gene modules MEmagenta, MEred and MEsalmon were positively associated with each pathological subtype: pathological amyloid, AD and LBD relative to controls, *q-*value <0.05 and Pearson's *r* > 0.1 (Figure 7A). When examining other variables including pathology scores, sex, age, brain weight, and the number of *APOE* ε4 alleles we observed that MEmagenta had the strongest association with age, *q*-value <0.001 and Pearson’s *r* = 0.32 (Fig. 7A). Modules MEpurple, MEgreen, MEblue and MEyellow, were positively associated with both AD and LBD and not shared with PA. Among the modules positively associated with both AD and LBD and not shared with PA, the MEgreen and MEblue were positively associated with Thal amyloid phase. The MEtan module was negatively associated with AD (*r* = −0.12) and Thal amyloid phase (*r* = −0.13). The MEpink module was associated with LBD versus control, LBD versus AD, and cingulate LB count (Fig. 7A).

We conducted an enrichment analysis to illuminate the functional implications for the identified gene modules (Supplementary Table 7). We performed GO analysis on the 50 genes within the cyan module, which displayed a negative association with both LBD compared to controls (*r* = −0.17) and LBD versus AD (*r* = −0.16). This analysis revealed significant enrichment for genes involved in ribosomal pathways (Fig. 7B). The pink module, encompassing 129 genes, displayed a positive association with LBD compared to control (*r* = 0.15), LBD versus AD (*r* = 0.13), and cingulate LB count (*r* = 0.19). GO analysis of this module highlighted enrichment for genes implicated in protein modification by small protein conjugation, including protein ubiquitination, epithelial to mesenchymal transition, negative regulation of phosphorylation and growth regulation (Fig. 7C). GO analysis of the 462 genes in the yellow module, which is positively associated with LBD versus control and AD versus control, was enriched for genes involved in the modulation of vesicle-mediated transport in synapse and nervous system development (Fig. 7D). A GO analysis of the 114 genes in the magenta module, positively associated with age, pathology and each disease type versus control, was enriched for immune response (Fig. 7E).

To refine the observed associations between the WGCNA modules and disease, we also implemented the WGNCA + DEGs approach as previously outlined.^37^ We observed that over 50% of the genes in the MEsalmon module were differentially expressed in the conventional gene-based comparative analysis for AD versus control and LBD versus controls (Supplementary Fig. 18). The MEsalmon module was enriched for genes related to response to protein folding and regulation of RNA splicing (Supplementary Fig. 17). Nearly 25% of the genes within the MEpurple are differentially expressed in the LBD versus control conventional gene-based comparative analysis (Supplementary Fig. 18). The MEpurple module was enriched for genes related to protein folding, RNA localization and chaperone-mediated protein complex assembly (Supplementary Fig. 17). Interestingly, we observed that less than 1% of the genes within MEpurple were differentially expressed in AD versus control comparison (Supplementary Fig. 18). These observations help further determine which genes and pathways are commonly and uniquely dysregulated in LBD and AD.

## Discussion

This study presents the most extensive Lewy body disease transcriptome analysis to date,^9-11,46^ encompassing 609 anterior cingulate cortex samples from a diverse cohort that includes individuals affected by LBD, AD and predominant amyloid-only pathology (PA), as well as control subjects. Our study pursued a two-fold objective: first, to evaluate alterations in gene expression patterns in each disease type relative to the control group, and second, to elucidate the shared and distinct gene alterations characterizing LBD and AD. We additionally stratified by sex and found many sex-specific differences in gene expression patterns, providing an understanding of the effect of sex within these neurodegenerative diseases. Finally, we employed a WGCNA to complement these aims. A subset of genes within these modules exhibited differential expression in the conventional gene-based comparative analysis. GO enrichment analysis for each set of differentially expressed genes and each identified WGCNA gene module strengthens our conclusions about shared and distinct pathways in each disease.

Neuroinflammation is believed to play a key role in the progression of clinical Lewy body dementia, AD and other related dementias.^46,47^ Specifically, it has been suggested that activated microglia are involved in the clearance of LB, debris and amyloid to maintain brain homeostasis^48,49^; however, over-activation may lead to increased production of pro-inflammatory molecules, resulting in excessive inflammation.^50-52^ In our study, genes dysregulated in the larger LBD group and the refined LBD subtypes (ATS and AS) compared to controls were strongly enriched in pathways related to inflammation, characterized by the robust activation of immune, cytokine and microglia-related genes, including activation/DAM genes.^41-43^ While not differentially expressed in the LBD (S) versus control, the activation/DAM microglia genes *SPP1*, *CSF1*, *TYROBP* and *TREM2* show upregulation, albeit not meeting the significance cut-off. This observation may be reflective of the early stage of the disease or less overall pathology compared to other groups. Even with the smaller sample size of the LBD (S) subtype compared to the larger LBD (ATS) and LBD (AS) groups, the LBD (S) comparison to the controls still reveals significant upregulation for genes involved in cell activation, cytokine signalling in the immune system and cellular response to cytokine stimulus. Our findings provide further evidence of the central role of neuroinflammation in the pathogenesis of LBD. Furthermore, we observed a substantial downregulation of genes associated with metabolic pathways, which could impair energy production and cellular homeostasis, thereby exacerbating neuronal dysfunction and degeneration.^46,53^ Lewy bodies accumulate within neurons, subsequently resulting in neuronal dysfunction and degeneration.^8^ Conversely, it may be that the neurons are deteriorating, and thus there is downregulation of genes involved in neuronal energy demand, as seen within ageing brains. In summary, the characterization of genes dysregulated in LBD compared to controls presented in this study has provided evidence of the upregulation of genes involved in neuroinflammation and the downregulation of genes involved in metabolic pathways.

To understand the transcriptional alterations observed in LBD, we stratified the LBD cases by diffuse (DLBD) and transitional (TLBD). In LBD, α-synuclein becomes misfolded and forms intraneuronal aggregates.^8^ Interestingly, protein folding emerged as a more significantly enriched pathway in TLBD cases, compared with DLBD and overall LBD. This observation was repeatedly observed in the LBD (S) cases, compared with LBD (AS) and LBD (ATS). These observations may point towards protective protein homeostasis pathways being engaged in response to the early accumulation of misfolded and aggregated α-synuclein protein.^54^ Furthermore, the direct comparison between DLBD and TLBD revealed the upregulation of genes involved in myelination, neurodevelopmental processes and cellular maintenance. DLBD represents a more advanced stage of LBD, and thus damage of myelin may contribute to compensatory mechanisms manifesting as upregulation of genes involved in myelination, potentially contributing to more advanced motor and cognitive deficits seen in the late stages of Lewy body dementia.^55,56^ Similarly, DLBD showed more significant upregulation of genes involved in cellular damage and dysfunction, including impaired cellular maintenance processes. The accumulation of misfolded proteins, including α-synuclein, may overwhelm cellular mechanisms responsible for protein clearance.^50^

Lewy body dementia is frequently misdiagnosed as AD.^57^ To better treat both Lewy body dementia and AD patients, it is critical to understand the molecular alterations common and unique to these neurodegenerative diseases. Commonly dysregulated pathways observed in both diseases were broadly enriched in the upregulation of inflammation and immune response modulation. Upregulated genes in these enriched inflammatory pathways included activation/DAM genes,^41-43^ such as *CSF1*, *TYROBP* and *TREM2*. *TREM2* (triggering receptor expressed on myeloid cells 2) encodes a receptor highly expressed in microglia. It has been suggested that *in vivo* inflammatory stimuli may be linked to increased *TREM2* expression, as reviewed by Jay *et al*.^57^ Activated microglia may be involved in the spread of pathologically misfolded proteins, as previous research has shown a correlation between microglial activation and Braak stages.^58^ It has also been suggested that activated microglia may be involved in removing toxic proteins to uphold brain homeostasis.^48,49^ Nonetheless, excessive activation can result in heightened production of pro-inflammatory molecules, thereby triggering inflammation.^50-52^ Furthermore, the VEGFA-VEGFR2 pathway, which influences vascular endothelial growth factor signalling, was also upregulated in LBD and AD, potentially impacting the integrity of the blood–brain barrier.^59^ Loss of blood–brain barrier integrity and vascular health, in general, is known to exacerbate neurodegenerative mechanisms.^60^ Commonly downregulated pathways in LBD and AD were involved in cellular and metabolic processes, including microtubule dynamics and neurotransmission, suggesting disrupted cellular communication. The common downregulation of metabolic pathways, particularly those related to mitochondrial function, may lead to oxidative stress and energy deficits, contributing to cell damage and neuronal death. Brain atrophy is a common feature of AD^61-63^ and some instances of LBD.^64^ These commonly dysregulated pathways in AD and LBD were mirrored in the WGCNA, where we observed a positive association between disease types in LBD and AD, and the gene modules MEmagenta and MEyellow that were enriched for immune and neuronal system pathways, respectively. These observations suggest a fundamental set of genes and pathways that respond to distinct neuropathological changes across diseases.

Synaptic signalling, behaviour and neuronal system pathways were downregulated in both LBD and AD; however, when directly comparing LBD to AD, these pathways were upregulated in LBD compared to AD. This observation is consistent with the extensive brain atrophy observed in AD patients, attributed to the loss and damage of neurons and shrinkage of the neuropil.^63^ Brain atrophy was also observed in the AD cases used for this study. A particularly striking finding was the pronounced downregulation of ribosomal genes in LBD versus AD as the result of greater gene expression of ribosomal genes in AD cases compared to the LBD cases. These observations were also seen in the AD versus control comparison, in which we observed significant upregulation of ribosomal genes, a phenomenon previously documented in brain capillaries of AD patients by Suzuki *et al*.^65^ The unique upregulation of ribosomal genes within AD versus LBD could be a compensatory mechanism to broadly upregulate protein synthesis in response to the loss of synaptic function and ongoing neuronal damage. We also identified a co-expression module enriched in genes predominantly linked to protein ubiquitination, which displayed a negative association with LBD but had no significant association with AD. This finding suggests an impairment of the ubiquitin-proteasome system (UPS), specifically in LBD, supporting a growing body of evidence that the development of Lewy-related pathology is influenced by dysfunctional misfolded protein degradation machinery.^66-68^ Altogether, these findings underscore pathways uniquely dysregulated within these two distinct neurodegenerative conditions, which may help point towards more specialized therapies.

Our investigation into sex differences in gene expression within LBD and AD yielded intriguing results. Despite a slightly smaller representation of females in our study cohort, we consistently observed a greater number of DEGs, and the overall magnitude of gene expression changes was more pronounced in XX females than XY males. The heightened gene dysregulation in females is consistent with previous studies that have indicated an upregulation of immune-related genes compared to males in AD.^69-72^ The sex chromosomes are the most sex-biased region of the genome,^73^ and several X-linked genes have been associated with sex differences in immune responses.^71^ In this study, we observed that most sex-differentially expressed genes were located on the X and Y chromosomes. Furthermore, we observed two X chromosome PAR1 genes (*GTPBP6* and *PPP2R3B*) significantly oppositely expressed between XX females and XY males, suggesting sex differences in gene dysregulation on the X chromosome within LBD. Several studies have suggested the potential role of the sex chromosomes in modulating neurodegenerative vulnerability.^70-75^ We observed significantly higher *KDM6A* expression, a gene that routinely escapes X inactivation,^76^ in XX females compared to XY males, and *KDM6A* expression has been associated with cognitive decline.^76,77^ Overall, our data highlights the sex-shared and sex-specific alterations in LBD and AD.

In conclusion, our comprehensive transcriptome analysis of LBD represents a significant step forward in understanding the molecular underpinnings of this complex neurodegenerative disease. By examining a diverse cohort of neuropathological types and control subjects, our study highlights the prominent role of neuroinflammation and metabolic dysfunction in LBD and AD pathogenesis, providing a framework for generalizing disease mechanisms. Moreover, our comparison of LBD subtypes by diffuse versus transitional Lewy pathology distribution reveals distinctive molecular states associated with the disease stage. To complement these findings, we characterized sex-specific and sex-differential gene expression to understand the effect of sex in these neurodegenerative diseases. Overall, these data underscore commonalities and differences in different neuropathologically defined types and serve as a resource to the community for new hypotheses.

### Limitations

The curation of the extensive LBD transcriptome dataset presented here represents a significant endeavour. Nevertheless, it is important to acknowledge the inherent limitations associated with such an undertaking. Tissues used in this study were sourced from the Mayo Clinic Florida brain bank, where clinical diagnoses of dementia, cause of death, post-mortem interval and a diverse representation of individuals from different population ancestries are not consistently available. Based on available clinical diagnoses within the brain bank database, most samples in this study would have presented with dementia as a clinical feature, but this information was not always available and was not performed by the same neurologist. On the other hand, pathology annotation was meticulously curated by a single neuropathologist (D.W.D); thus, this study focused on neuropathologically defined cases. The selection of the anterior cingulate cortex region for analysis was based on its critical involvement in cognitive processes. While incorporating multiple tissue regions from each individual would have provided valuable insights into transcriptional alterations regarding disease progression, the large sample size of 609 individuals rendered this approach unfeasible. Efforts were made to provide a comprehensive overview of transcriptional alterations among neuropathologically defined cases, focusing primarily on gene-level alterations rather than isoform dysregulation. Future investigations could delve into characterizing isoform differences to elucidate further molecular mechanisms underlying LBD. While alternative models may exist for sub-analyses, a consistent approach was employed throughout this study to facilitate comparability between different comparisons. Nonetheless, this large transcriptome dataset of neuropathologically defined groups is of great value to the scientific community to better understand the molecular alterations that are shared or uniquely dysregulated between LBD and AD.

## Supplementary Material

awae202_Supplementary_Data

## Data Availability

Data are deposited at Short Read Archive (SRA) BioProject PRJNA1023207. This study's processing pipeline is available at https://github.com/fryerlab/LBD_CWOW and includes a reproducible conda environment. Interactive link to query genes: https://fryerlab.shinyapps.io/LBD_CWOW/. Processed counts tables are available at synapse.org; SnyID: syn52394100.
