# Supplementary material for: Distinct transcriptional alterations distinguish Lewy body disease from Alzheimer’s disease
Source: Brain. 2024 Jun 25;148(1):69–88. doi: 10.1093/brain/awae202 (PMC11706328; doi:10.1093/brain/awae202)
Supplement: awae202_Supplementary_Data [file awae202_supplementary_data.zip › brain-2023-02363-File010.pdf]

Supplementary Table 1. Metadata. Sample information includes neuropathically defined disease type Lewy body disease (LBD), Alzheimer's disease (AD), pathological amyloid (PA) consisting of amyloid pathology with minimal or no tau pathology, and control. Clinical diagnosis, sample ID (NPID), pathology scores, APOE genotype, brain weight, age at death, sex inferred from RNAseq expression, reported race, postmortem interval (PMI), RNA integrity number (RIN), and flowcell.

Supplementary Table 2. Differentially expressed genes for each pairwise comparison. Genes are differentially expressed if the adjusted *p*-value is < 0.05 and absolute $\log_2 FC > 0.25$. Columns contain chromosome (seqnames) and gene information, the $\log_2$FC, along with the 95% confidence interval (CI). Also reported is the average expression across all samples ($\log_2$CPM); t, which is logFC divided by its standard error; *p*-value (based on t) from a test that logFC differs from 0; adj.P.Val Benjamini-Hochberg false discovery rate (*q*-value); and B log-odds that the gene is differentially expressed.

Supplementary Table 3. Metascape gene ontology enrichment analysis for up and down-regulated genes for each pairwise comparison. Enrichment analysis was performed individually for up and down-regulated genes within each comparison utilizing metascape.org. Enriched gene ontology terms are clustered into summaries. Log *p*-value and *q*-value are reported for each term, along with the corresponding gene symbols within each term.

Supplementary Table 4. Multi-gene list Metascape gene ontology enrichment analysis for up or down-regulated genes among disease types. Enrichment analysis was performed jointly among disease types for up and repeated for down-regulated genes utilizing Metascape.org multi-

gene list analysis. Enriched gene ontology terms are clustered into summaries. Log *p*-value and *q*-value are reported for each term, along with the corresponding gene symbols within each term.

Supplementary Table 5. Differentially expressed genes within each sex for each pairwise comparison. Genes are differentially expressed if the adjusted *p*-value is < 0.05 and absolute $log_2FC > 0.25$. Columns contain chromosome (seqnames) and gene information, the log_2FC along with the 95% confidence interval (CI). Also reported is the average expression across all samples (log_2CPM); t, which is logFC divided by its standard error; *p*-value (based on t) from the test that logFC differs from 0; adj.P.Val Benjamini-Hochberg false discovery rate; and B log-odds that the gene is differentially expressed.

Supplementary Table 6. Weighted Gene Correlation Network Analysis (WGCNA) gene modules. Genes within each WGCNA gene module.

Supplementary Table 7. Metascape GO enrichment analysis for WGCNA gene modules. Enrichment analysis was performed for each WGCNA gene module. Enriched GO terms are clustered into summaries. Log *p*-value and *q*-value are reported for each term, along with the corresponding gene symbols within each term.

a)

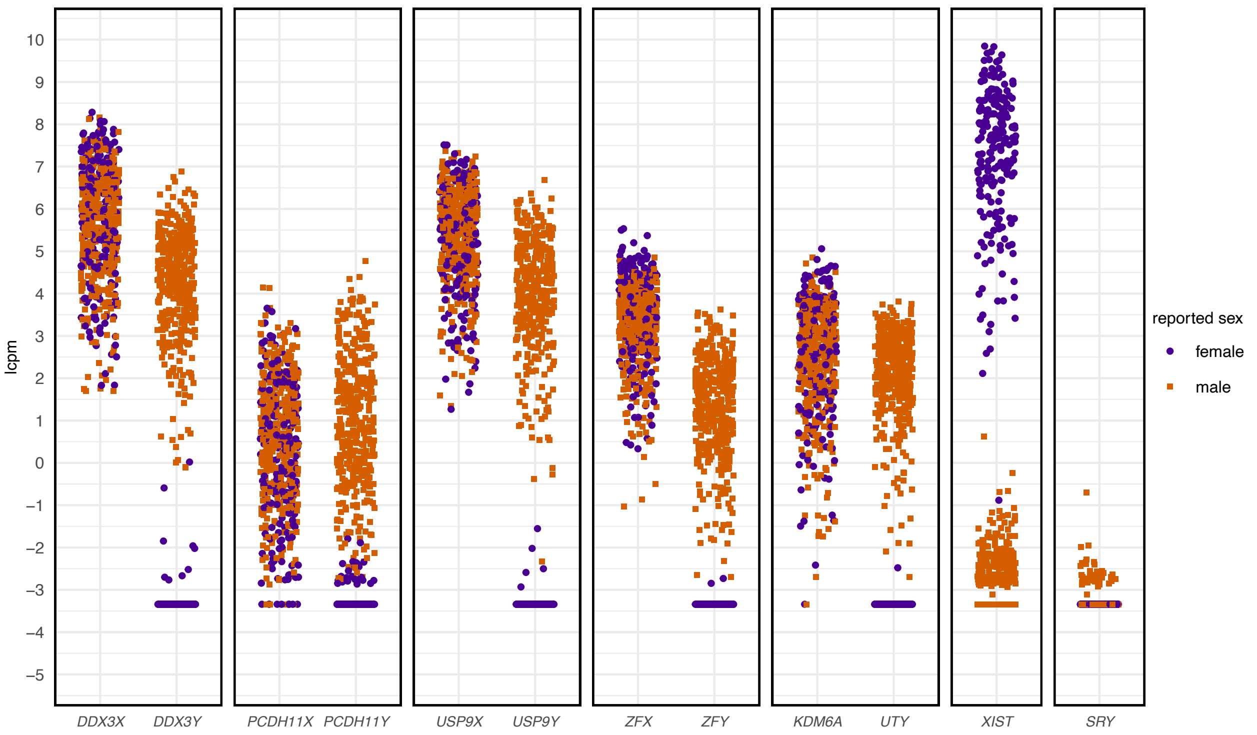

lcpm
10
9
8
7
6
5
4
3
2
1
0
−1
−2
−3
−4
−5
DDX3X
DDX3Y
PCDH11X
PCDH11Y
USP9X
USP9Y
ZFX
ZFY
KDM6A
UTY
XIST
SRY
reported sex
female
male

b)

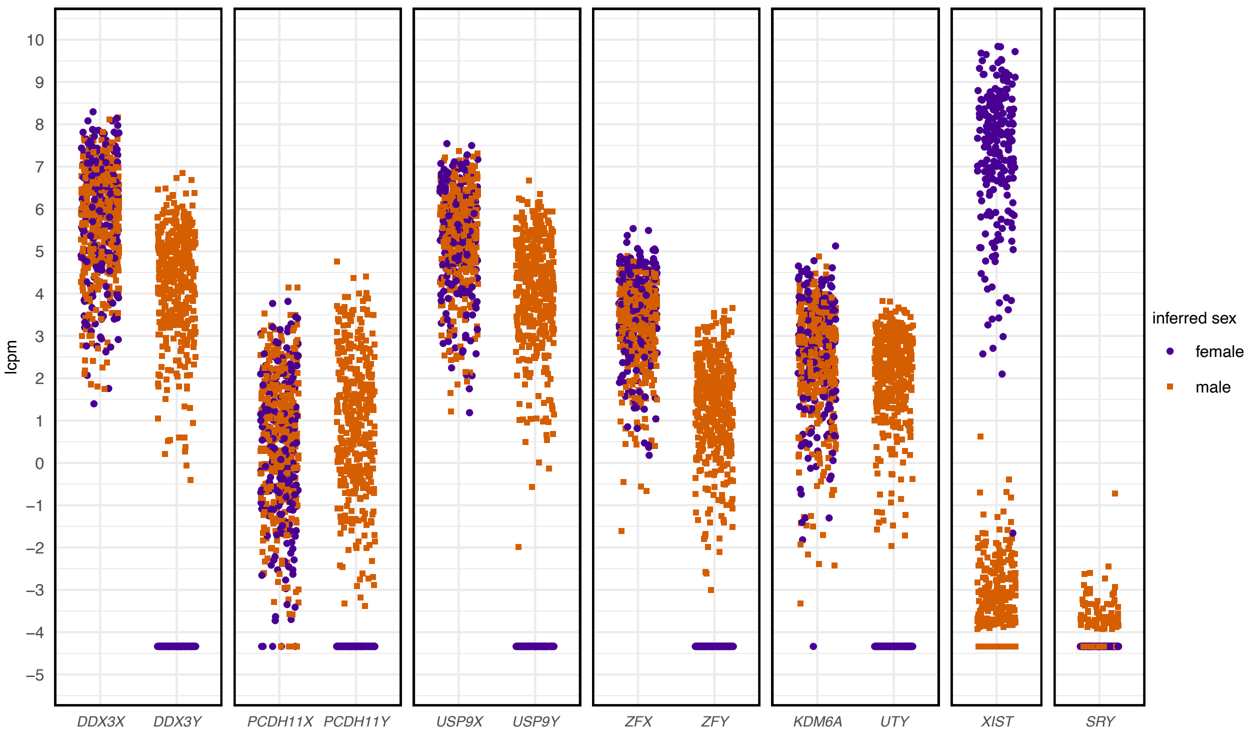

lcpm
10
9
8
7
6
5
4
3
2
1
0
−1
−2
−3
−4
−5
DDX3X
DDX3Y
PCDH11X
PCDH11Y
USP9X
USP9Y
ZFX
ZFY
KDM6A
UTY
XIST
SRY
inferred sex
female
male

Supplementary Fig. 1. Sample sex check. a) Samples were first aligned to a default reference genome that includes the full sequences of both the X and Y chromosomes. The sex chromosome complement of the sample was checked by investigating the expression of five X-Y homologous genes (*EIF1AY*, *KDM5D*, *UTY*, *DDX3Y*, *RPS4Y1*), one X-linked gene (*XIST*), and one Y-linked gene (*SRY*). **b)** Samples were then re-aligned to a reference genome informed on the sex chromosome complement.

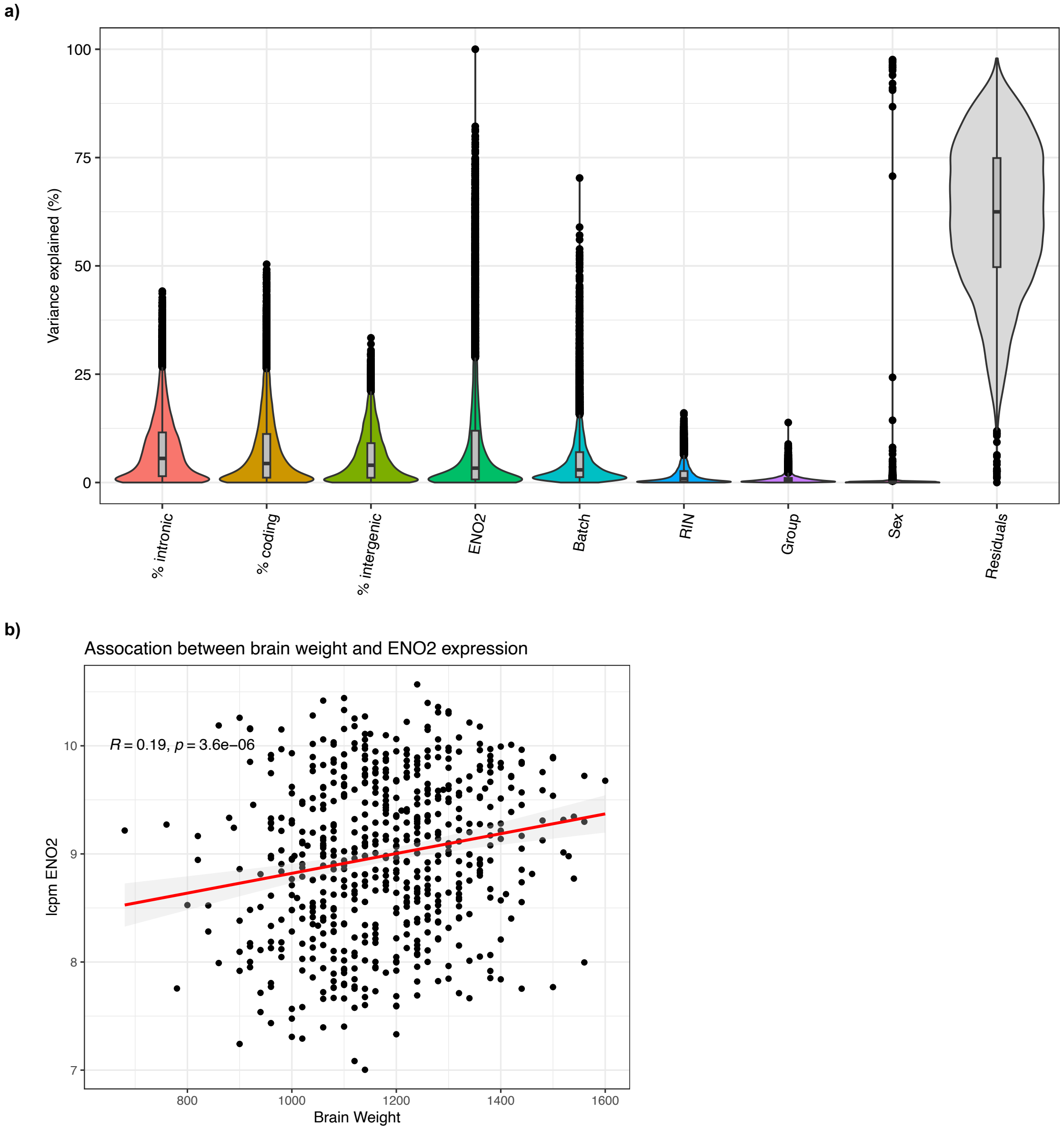

Supplementary Fig. 2. Assessment of variation in gene expression. a) Genome-wide violin plot of the distribution of variance explained by each variable across all genes, ranked by the total contribution. **b)** Positive association between brain weight, measured in grams, and *ENO2* expression measured in $\log_2$ count per million (cpm).

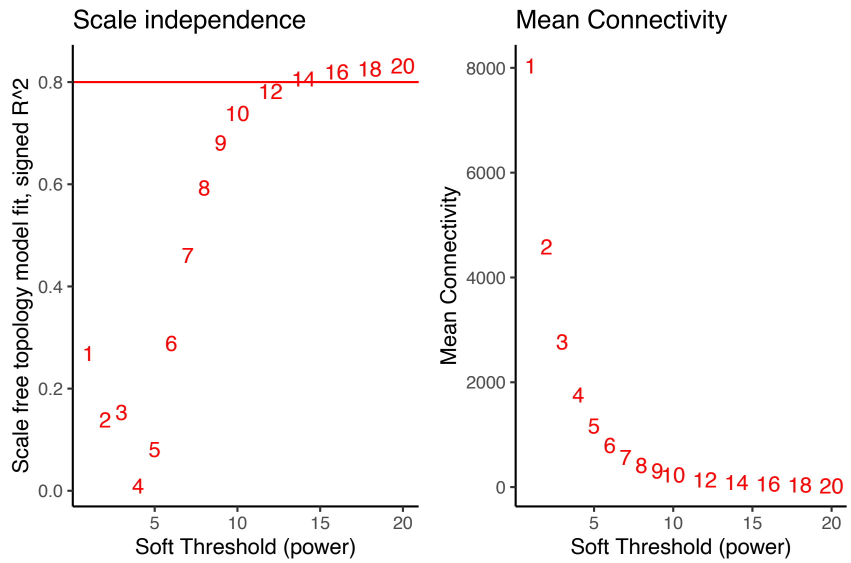

Supplementary Fig. 3. Power selection for Weighted Gene Correlation Network Analysis (WGNCA). Soft-thresholding powers 1 through 20 were tested to determine a power that gives a scale-free topology. Here, a soft threshold power 12 was selected to achieve ~80% signed R^2 of the model fit.

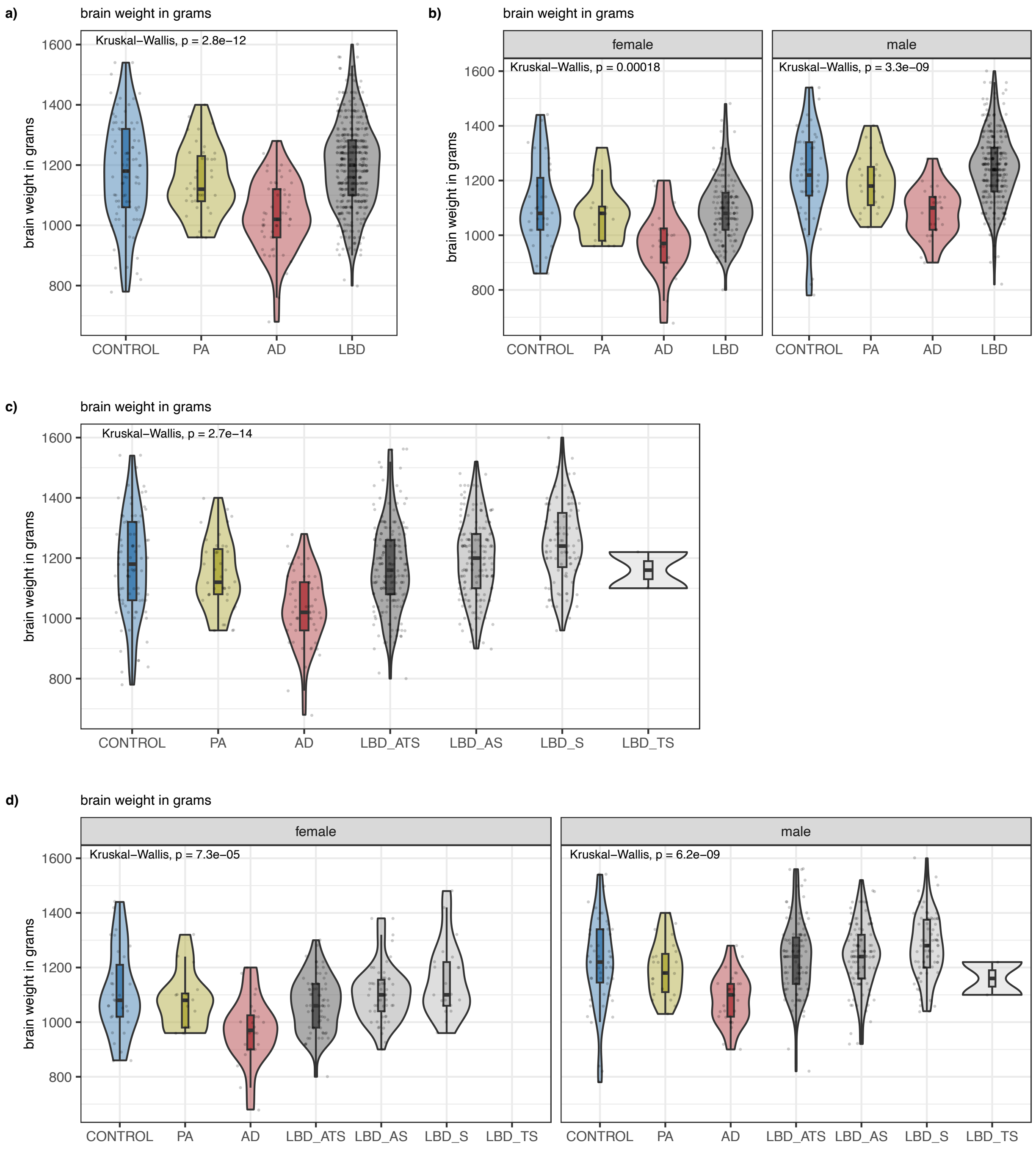

Supplementary Fig. 4. Brain weight among disease types. a) Brain weight, measured in grams, significantly differs among disease types in control (*n* = 81), pathological amyloid cases consisting of amyloid pathology with minimal or no tau pathology (PA, *n* = 39), Alzheimer's disease (AD, *n* = 53), and Lewy body disease (LBD, *n* = 436). The lowest median brain weight

was observed in Alzheimer's disease (AD) cases (H(3) = 56.8, *p*-value < 0.001). **b)** Brain weight within XX female cases (left) and XY male cases (right) are significantly different among disease types. **c) – d)** When the Lewy body disease (LBD) cases were further stratified into distinct cohorts based on neuropathological criteria (ATS, AS, S, TS), we again observed significant differences in brain weight among the disease types (H(6) = 75.7, *p*-value < 0.001).

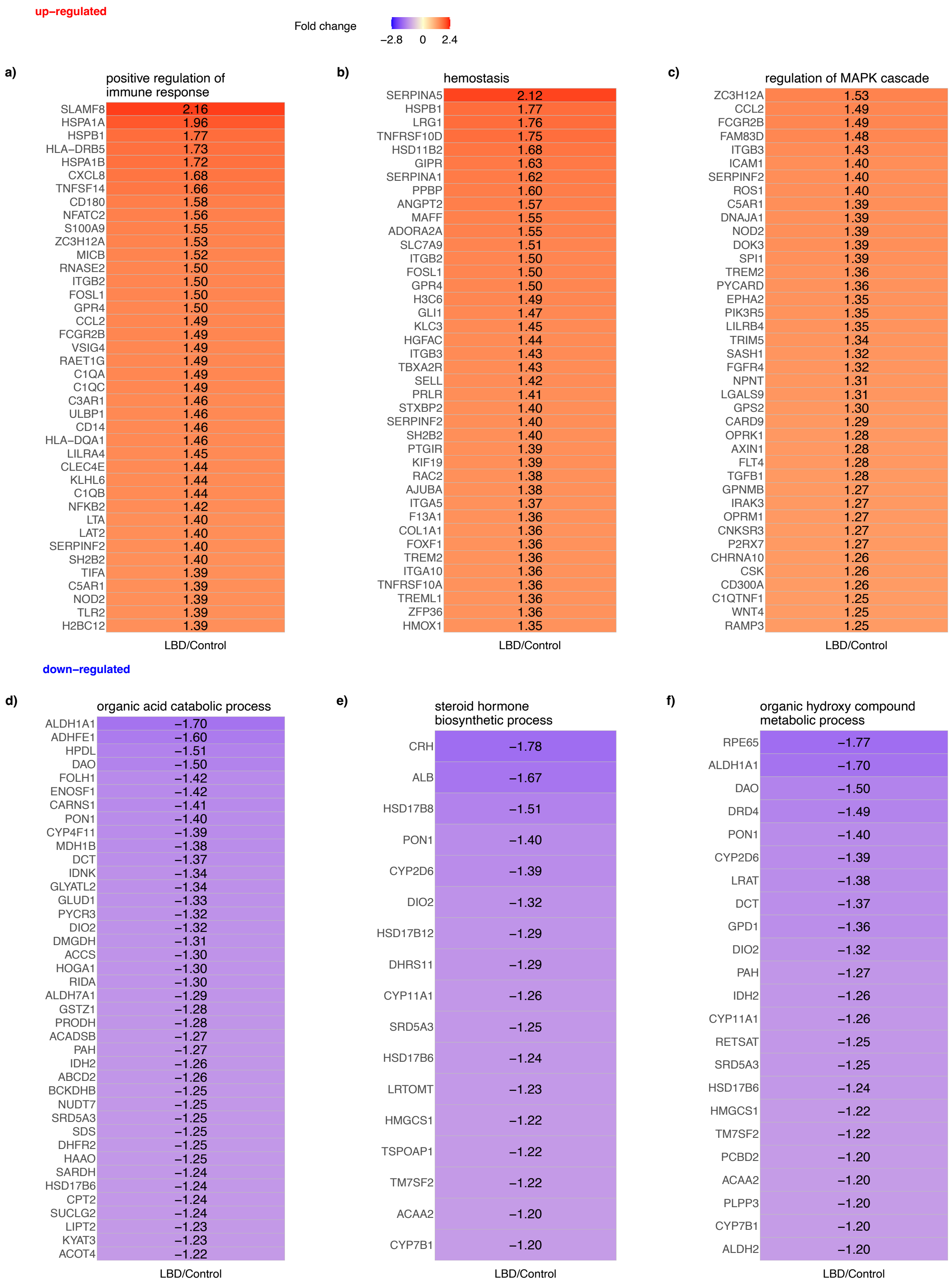

up−regulated
Fold change
−2.8
0
2.4
a)
positive regulation of immune response
SLAMF8 2.16
HSPA1A 1.96
HSPB1 1.77
HLA−DRB5 1.73
HSPA1B 1.72
CXCL8 1.68
TNFSF14 1.66
CD180 1.58
NFATC2 1.56
S100A9 1.55
ZC3H12A 1.53
MICB 1.52
RNASE2 1.50
ITGB2 1.50
FOSL1 1.50
GPR4 1.50
CCL2 1.49
FCGR2B 1.49
VSIG4 1.49
RAET1G 1.49
C1QA 1.49
C1QC 1.49
C3AR1 1.46
ULBP1 1.46
CD14 1.46
HLA−DQA1 1.46
LILRA4 1.45
CLEC4E 1.44
KLHL6 1.44
C1QB 1.44
NFKB2 1.42
LTA 1.40
LAT2 1.40
SERPINF2 1.40
SH2B2 1.40
TIFA 1.39
C5AR1 1.39
NOD2 1.39
TLR2 1.39
H2BC12 1.39
LBD/Control
b)
hemostasis
SERPINA5 2.12
HSPB1 1.77
LRG1 1.76
TNFRSF10D 1.75
HSD11B2 1.68
GIPR 1.63
SERPINA1 1.62
PPBP 1.60
ANGPT2 1.57
MAFF 1.55
ADORA2A 1.55
SLC7A9 1.51
ITGB2 1.50
FOSL1 1.50
GPR4 1.50
H3C6 1.49
GLI1 1.47
KLC3 1.45
HGFAC 1.44
ITGB3 1.43
TBXA2R 1.43
SELL 1.42
PRLR 1.41
STXBP2 1.40
SERPINF2 1.40
SH2B2 1.40
PTGIR 1.39
KIF19 1.39
RAC2 1.38
AJUBA 1.38
ITGA5 1.37
F13A1 1.36
COL1A1 1.36
FOXF1 1.36
TREM2 1.36
ITGA10 1.36
TNFRSF10A 1.36
TREML1 1.36
ZFP36 1.36
HMOX1 1.35
LBD/Control
c)
regulation of MAPK cascade
ZC3H12A 1.53
CCL2 1.49
FCGR2B 1.49
FAM83D 1.48
ITGB3 1.43
ICAM1 1.40
SERPINF2 1.40
ROS1 1.40
C5AR1 1.39
DNAJA1 1.39
NOD2 1.39
DOK3 1.39
SPI1 1.39
TREM2 1.36
PYCARD 1.36
EPHA2 1.35
PIK3R5 1.35
LILRB4 1.35
TRIM5 1.34
SASH1 1.32
FGFR4 1.32
NPNT 1.31
LGALS9 1.31
GPS2 1.30
CARD9 1.29
OPRK1 1.28
AXIN1 1.28
FLT4 1.28
TGFB1 1.28
GPNMB 1.27
IRAK3 1.27
OPRM1 1.27
CNKSR3 1.27
P2RX7 1.27
CHRNA10 1.26
CSK 1.26
CD300A 1.26
C1QTNF1 1.25
WNT4 1.25
RAMP3 1.25
LBD/Control
down−regulated
d)
organic acid catabolic process
ALDH1A1 −1.70
ADHFE1 −1.60
HPDL −1.51
DAO −1.50
FOLH1 −1.42
ENOSF1 −1.42
CARNS1 −1.41
PON1 −1.40
CYP4F11 −1.39
MDH1B −1.38
DCT −1.37
IDNK −1.34
GLYATL2 −1.34
GLUD1 −1.33
PYCR3 −1.32
DIO2 −1.32
DMGDH −1.31
ACCS −1.30
HOGA1 −1.30
RIDA −1.30
ALDH7A1 −1.29
GSTZ1 −1.28
PRODH −1.28
ACADSB −1.27
PAH −1.27
IDH2 −1.26
ABCD2 −1.26
BCKDHB −1.25
NUDT7 −1.25
SRD5A3 −1.25
SDS −1.25
DHFR2 −1.25
HAAO −1.25
SARDH −1.24
HSD17B6 −1.24
CPT2 −1.24
SUCLG2 −1.24
LIPT2 −1.23
KYAT3 −1.23
ACOT4 −1.22
LBD/Control
e)
steroid hormone biosynthetic process
CRH −1.78
ALB −1.67
HSD17B8 −1.51
PON1 −1.40
CYP2D6 −1.39
DIO2 −1.32
HSD17B12 −1.29
DHRS11 −1.29
CYP11A1 −1.26
SRD5A3 −1.25
HSD17B6 −1.24
LRTOMT −1.23
HMGCS1 −1.22
TSPOAP1 −1.22
TM7SF2 −1.22
ACAA2 −1.20
CYP7B1 −1.20
LBD/Control
f)
organic hydroxy compound metabolic process
RPE65 −1.77
ALDH1A1 −1.70
DAO −1.50
DRD4 −1.49
PON1 −1.40
CYP2D6 −1.39
LRAT −1.38
DCT −1.37
GPD1 −1.36
DIO2 −1.32
PAH −1.27
IDH2 −1.26
CYP11A1 −1.26
RETSAT −1.25
SRD5A3 −1.25
HSD17B6 −1.24
HMGCS1 −1.22
TM7SF2 −1.22
PCBD2 −1.20
ACAA2 −1.20
PLPP3 −1.20
CYP7B1 −1.20
ALDH2 −1.20
LBD/Control

Supplementary Fig. 5. Lewy body disease versus control Metascape gene heatmaps. Gene heatmaps compare LBD (Lewy body disease) samples to control samples. The heatmaps illustrate the fold change values for the top 40 genes associated with selected up-regulated and down-regulated gene ontology terms.

a) **up–regulated**

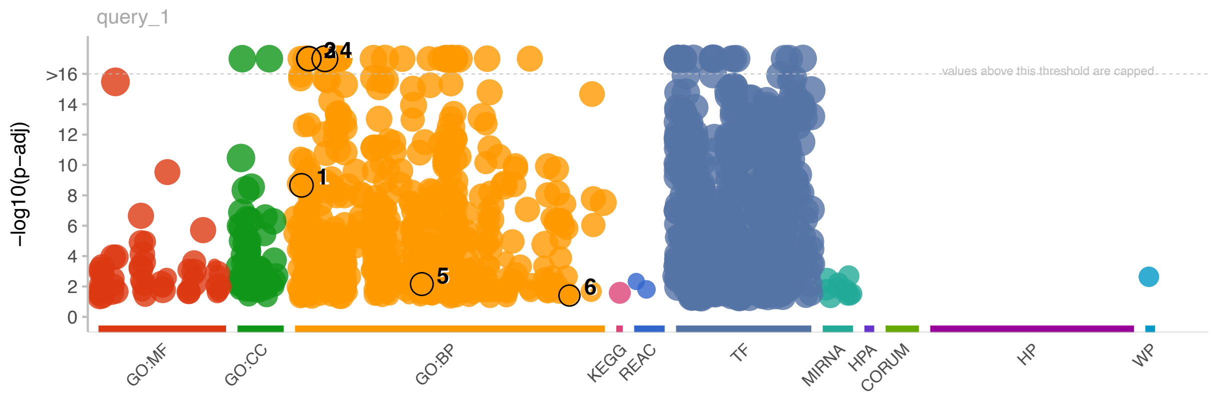

id	source	term_id	term_name	intersection_size		p_value
1	GO:BP	GO:0001944	vasculature development	82		2.2e−09
2	GO:BP	GO:0002684	positive regulation of immune system process	127		2.2e−20
3	GO:BP	GO:0002682	regulation of immune system process	165		1.1e−22
4	GO:BP	GO:0006950	response to stress	301		1.3e−18
5	GO:BP	GO:0043549	regulation of kinase activity	46		6.9e−03
6	GO:BP	GO:1904018	positive regulation of vasculature development	20		3.9e−02

g:Profiler (biit.cs.ut.ee/gprofiler)

b) **down–regulated**

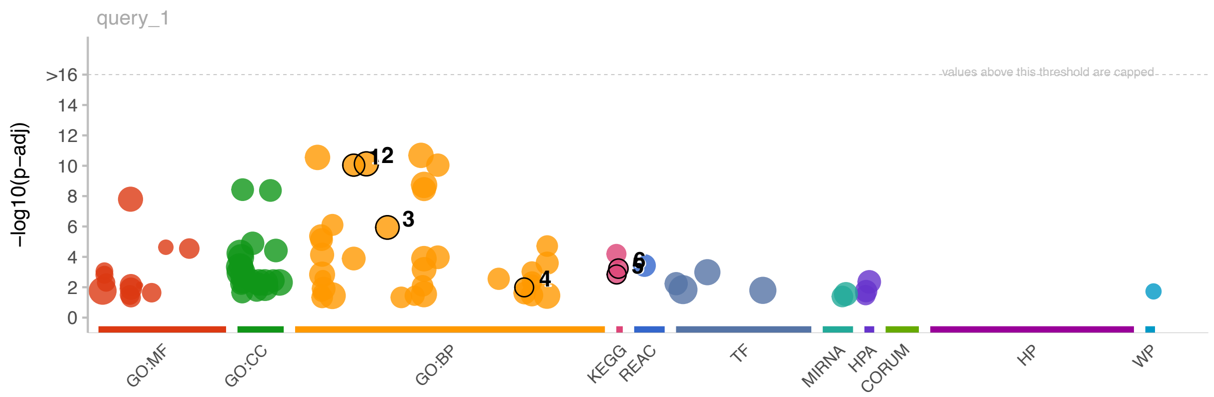

id	source	term_id	term_name	intersection_size		p_value
1	GO:BP	GO:0016054	organic acid catabolic process	32		9.1e−11
2	GO:BP	GO:0019752	carboxylic acid metabolic process	64		7.5e−11
3	GO:BP	GO:0032787	monocarboxylic acid metabolic process	24		1.2e−06
4	GO:BP	GO:0120178	steroid hormone biosynthetic process	8		1.0e−02
5	KEGG	KEGG:00280	Valine, leucine and isoleucine degradation	8		1.4e−03
6	KEGG	KEGG:01212	Fatty acid metabolism	8		5.9e−04

g:Profiler (biit.cs.ut.ee/gprofiler)

Supplementary Fig. 6. Manhattan plot of Lewy body disease versus control g:Profiler enrichment analysis. a) up-regulated and **b)** down-regulated enrichment terms. The x-axis of the plot corresponds to functional terms, which are grouped and color-coded based on data sources. The y-axis represents the adjusted enrichment -log10 p-values. The p-values are capped at 10^-16. The size of the circle is proportional to the size of the corresponding functional term. The table highlights selected gene ontology terms.

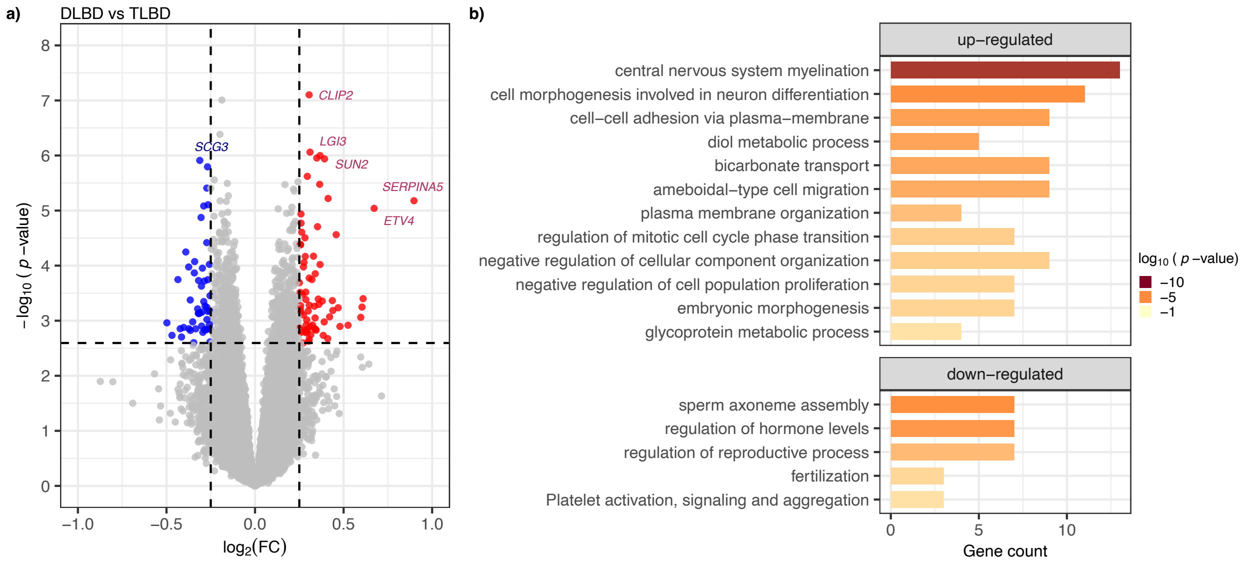

Supplementary Fig. 7. Diffuse versus transitional Lewy body disease. a) Gene differential expression between diffuse Lewy body disease (DLBD, n = 339) compared to transitional Lewy body disease (TLBD, n = 95). **b)** Differentially expressed genes in DLBD versus TLBD are enriched in central nervous system myelination and cell morphogenesis.

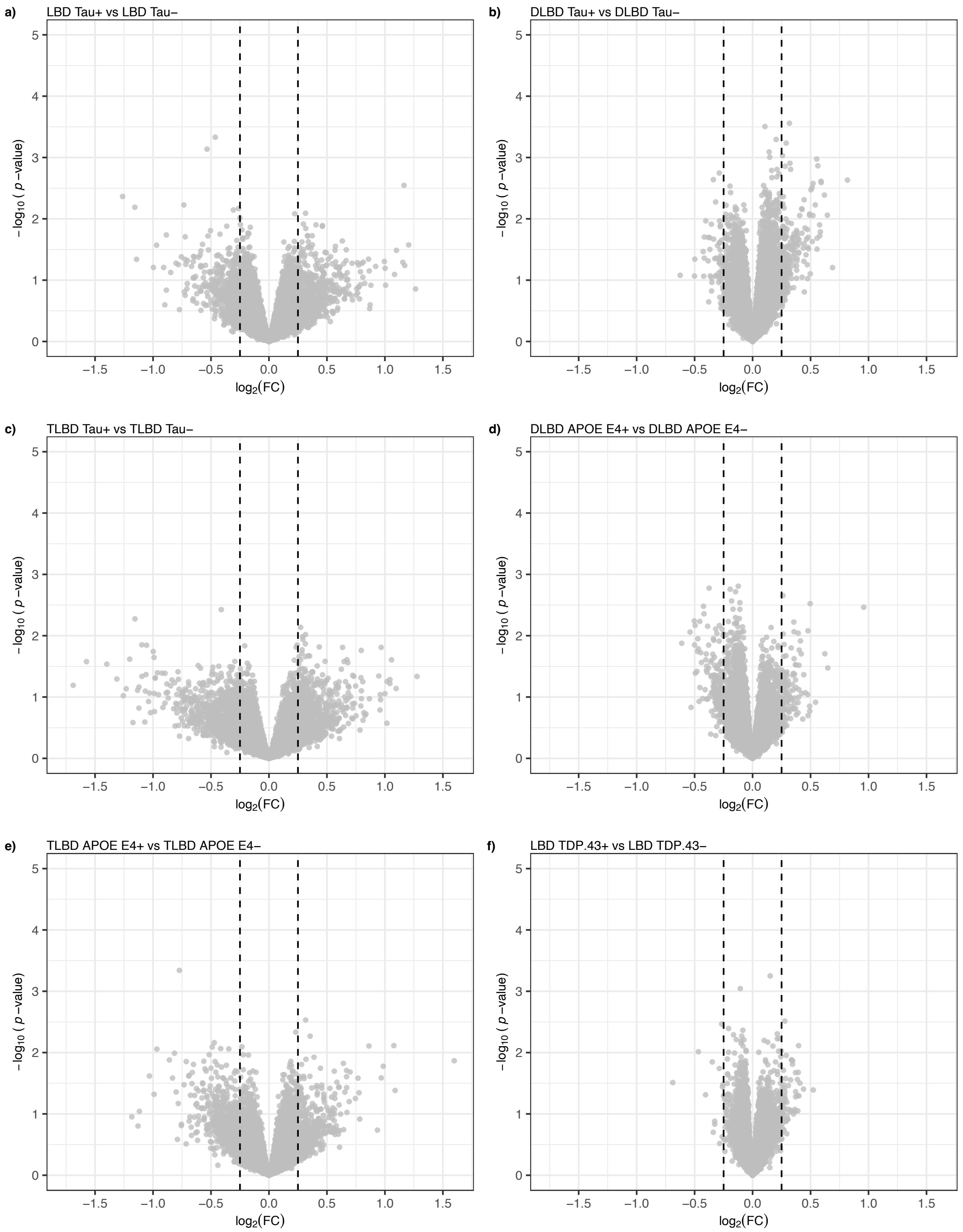

a)
LBD Tau+ vs LBD Tau−
b)
DLBD Tau+ vs DLBD Tau−
c)
TLBD Tau+ vs TLBD Tau−
d)
DLBD APOE E4+ vs DLBD APOE E4−
e)
TLBD APOE E4+ vs TLBD APOE E4−
f)
LBD TDP.43+ vs LBD TDP.43−
−log10 (p −value)
log2(FC)
−1.5
−1.0
−0.5
0.0
0.5
1.0
1.5
0
1
2
3
4
5

Supplementary Fig. 8. Tau, *APOE* ε4 positive, or TPD-43 positive don't contribute to differential gene expression within Lewy body disease cases. There are no significant differentially expressed genes between tau positive as defined by Braak NFT equal and greater than IV and tau negative cases with a Braak NFT stage less or equal to III within **a)** Lewy body disease (Tau+ n = 180 vs. Tau- n = 256), **b)** diffuse Lewy body disease (Tau+ n = 171 vs. Tau- n = 168), or **c)** transitional Lewy body disease (Tau+ n = 9 vs. Tau- n = 86). There are no significant differentially expressed genes between *APOE* ε4 positive compared to *APOE* ε4 negative cases within **d)** diffuse Lewy body disease (ε4+ n = 188 vs. ε4- n = 151), nor in **e)** transitional Lewy body disease (ε4+ n = 19 vs. ε4- n = 76). **f)** Comparison between Lewy body disease TDP-43 positive (n = 134) versus Lewy body disease TDP-43 negative (n = 289) cases revealed no differentially expressed genes.

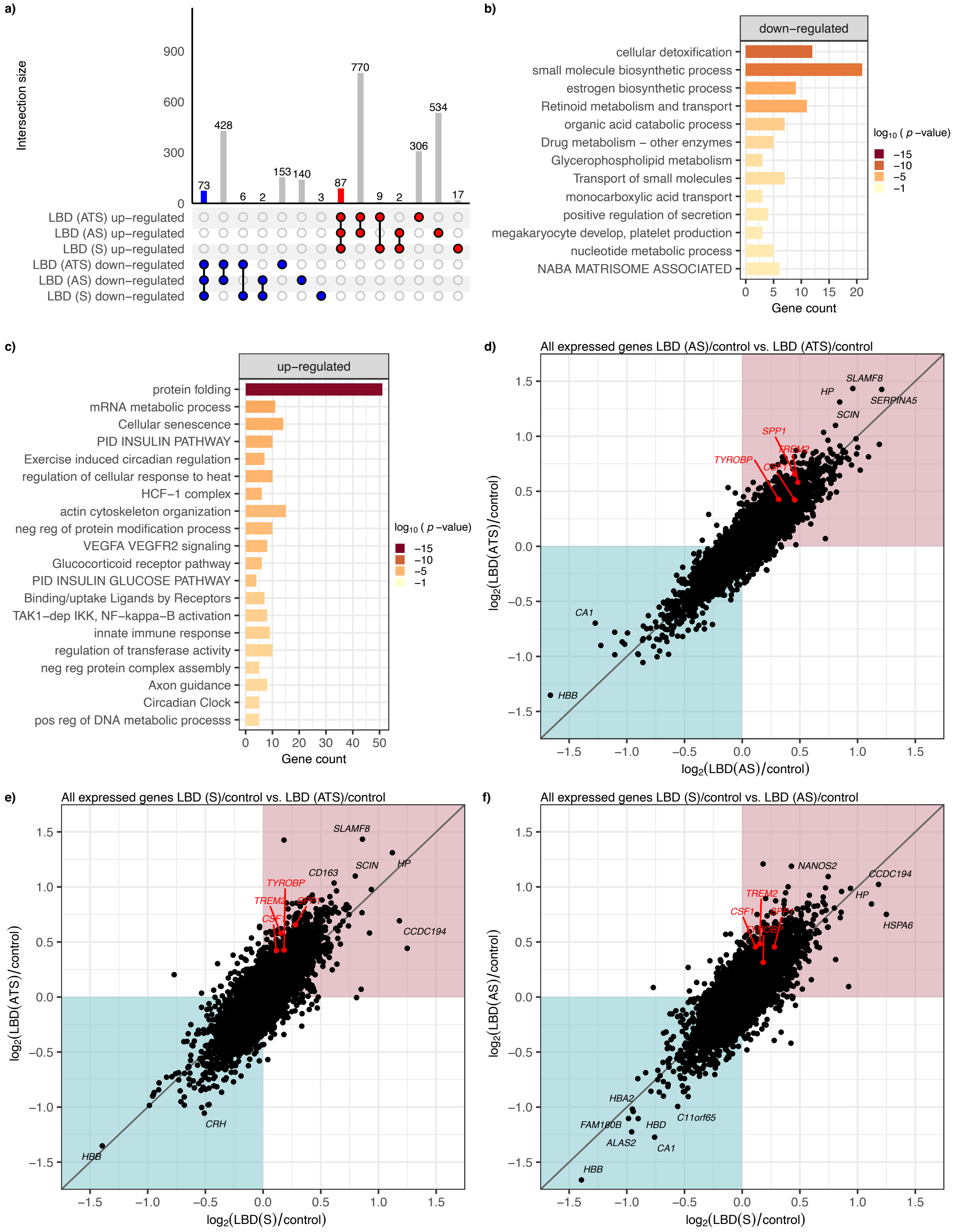
a)
Intersection size
0
300
600
900
73
428
6
2
153
140
3
87
770
9
2
306
534
17
LBD (ATS) up−regulated
LBD (AS) up−regulated
LBD (S) up−regulated
LBD (ATS) down−regulated
LBD (AS) down−regulated
LBD (S) down−regulated
b)
down−regulated
cellular detoxification
small molecule biosynthetic process
estrogen biosynthetic process
Retinoid metabolism and transport
organic acid catabolic process
Drug metabolism − other enzymes
Glycerophospholipid metabolism
Transport of small molecules
monocarboxylic acid transport
positive regulation of secretion
megakaryocyte develop, platelet production
nucleotide metabolic process
NABA MATRISOME ASSOCIATED
Gene count
0
5
10
15
20
log10 (p −value)
−15
−10
−5
−1
c)
up−regulated
protein folding
mRNA metabolic process
Cellular senescence
PID INSULIN PATHWAY
Exercise induced circadian regulation
regulation of cellular response to heat
HCF−1 complex
actin cytoskeleton organization
neg reg of protein modification process
VEGFA VEGFR2 signaling
Glucocorticoid receptor pathway
PID INSULIN GLUCOSE PATHWAY
Binding/uptake Ligands by Receptors
TAK1−dep IKK, NF−kappa−B activation
innate immune response
regulation of transferase activity
neg reg protein complex assembly
Axon guidance
Circadian Clock
pos reg of DNA metabolic processs
Gene count
0
10
20
30
40
50
log10 (p −value)
−15
−10
−5
−1
d)
All expressed genes LBD (AS)/control vs. LBD (ATS)/control
log2(LBD(ATS)/control)
log2(LBD(AS)/control)
SLAMF8
HP
SERPINA5
SCIN
SPP1
TREM2
TYROBP
CSF1
CA1
HBB
e)
All expressed genes LBD (S)/control vs. LBD (ATS)/control
log2(LBD(ATS)/control)
log2(LBD(S)/control)
SLAMF8
SCIN
HP
CD163
TYROBP
TREM2
SPP1
CSF1
CCDC194
CRH
HBB
f)
All expressed genes LBD (S)/control vs. LBD (AS)/control
log2(LBD(AS)/control)
log2(LBD(S)/control)
NANOS2
CCDC194
HP
HSPA6
TREM2
CSF1
SPP1
TYROBP
HBA2
FAM180B
HBD
C11orf65
ALAS2
CA1
HBB
−1.5
−1.0
−0.5
0.0
0.5
1.0
1.5

Supplementary Fig. 9. Up-regulation of genes involved in protein folding and circadian regulation observed in Lewy body disease (LBD) versus control cases. a) The UpSet plot shows the number of shared and unique differentially expressed genes (DEGs) between Lewy body disease (ATS), LBD (AS), and LBD (S) relative to controls. **b)** Gene Ontology (GO) analysis of the 73 commonly down-regulated genes among the comparisons were enriched for cellular detoxification and biosynthetic process. The x-axis is the gene count contributing to the enrichment pathways listed on the y-axis. The color of the bar indicates the -$\log_{10}$ *p*-value. **c)** GO analysis of the 87 commonly up-regulated genes were enriched for protein folding and circadian regulation. **d)** Scatter plot of the $\log_2$FC of LBD (AS) versus control (x-axis) and LBD (ATS) versus control genes (y-axis), for all expressed genes (q-value ≤ 1), regardless of fold change direction or significance value. Genes within the bottom left teal box had a negative $\log_2$FC in both LBD (AS) and LBD (ATS) relative to controls, and genes within the top right pink box had a positive $\log_2$FC in both. Genes: *TYROBP*, *SPP1*, *TREM2*, and *CSF1* are shown in red. Repeated for the $\log_2$FC of **e)** LBD (S) relative to controls (x-axis) and LBD (ATS) versus control genes (y-axis) and **f)** LBD (S) relative to controls (x-axis) and LBD (AS) versus control genes (y-axis).

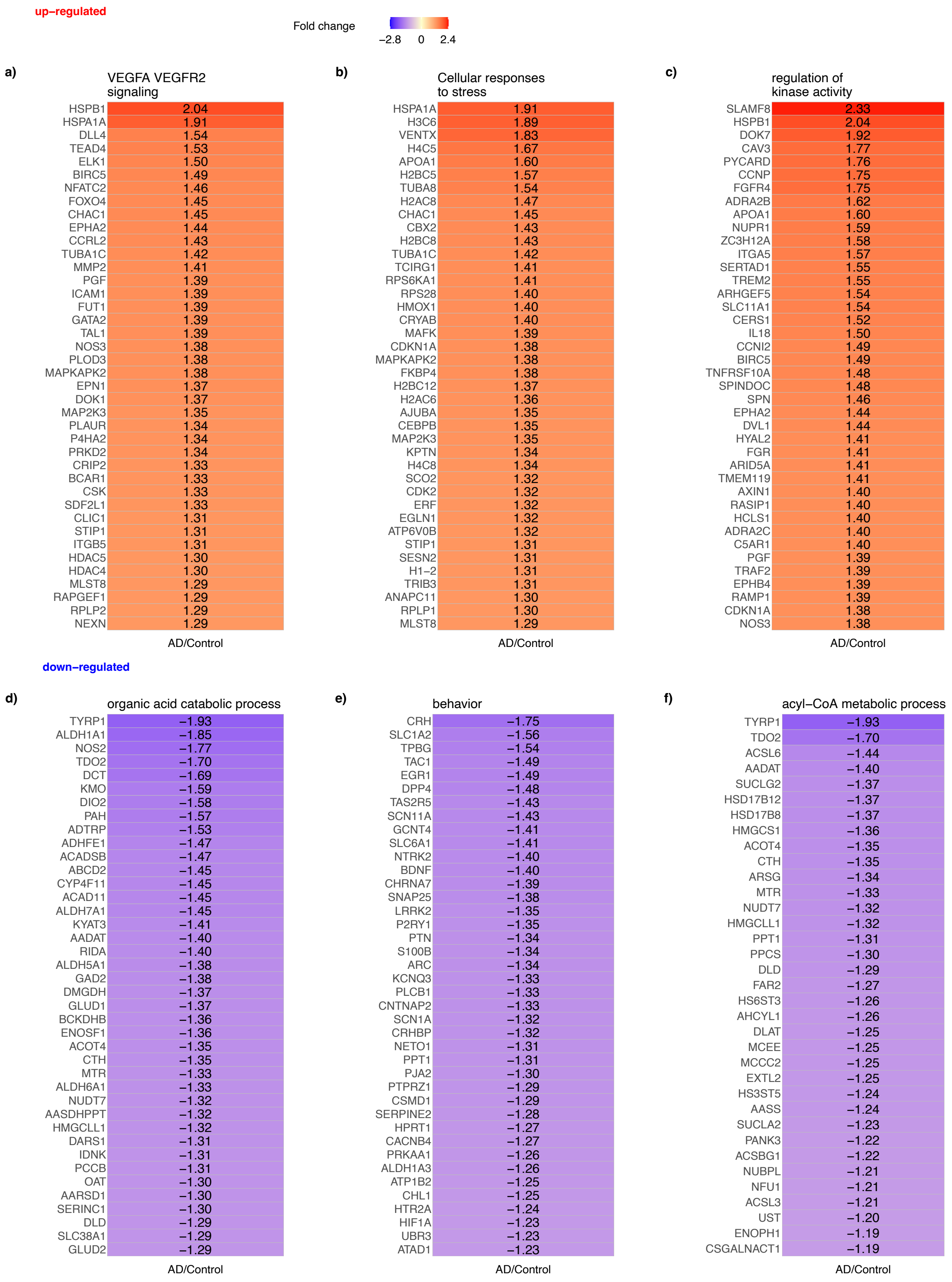

up−regulated
Fold change
−2.8
0
2.4
a)
VEGFA VEGFR2 signaling
HSPB1 2.04
HSPA1A 1.91
DLL4 1.54
TEAD4 1.53
ELK1 1.50
BIRC5 1.49
NFATC2 1.46
FOXO4 1.45
CHAC1 1.45
EPHA2 1.44
CCRL2 1.43
TUBA1C 1.42
MMP2 1.41
PGF 1.39
ICAM1 1.39
FUT1 1.39
GATA2 1.39
TAL1 1.39
NOS3 1.38
PLOD3 1.38
MAPKAPK2 1.38
EPN1 1.37
DOK1 1.37
MAP2K3 1.35
PLAUR 1.34
P4HA2 1.34
PRKD2 1.34
CRIP2 1.33
BCAR1 1.33
CSK 1.33
SDF2L1 1.33
CLIC1 1.31
STIP1 1.31
ITGB5 1.31
HDAC5 1.30
HDAC4 1.30
MLST8 1.29
RAPGEF1 1.29
RPLP2 1.29
NEXN 1.29
AD/Control
b)
Cellular responses to stress
HSPA1A 1.91
H3C6 1.89
VENTX 1.83
H4C5 1.67
APOA1 1.60
H2BC5 1.57
TUBA8 1.54
H2AC8 1.47
CHAC1 1.45
CBX2 1.43
H2BC8 1.43
TUBA1C 1.42
TCIRG1 1.41
RPS6KA1 1.41
RPS28 1.40
HMOX1 1.40
CRYAB 1.40
MAFK 1.39
CDKN1A 1.38
MAPKAPK2 1.38
FKBP4 1.38
H2BC12 1.37
H2AC6 1.36
AJUBA 1.35
CEBPB 1.35
MAP2K3 1.35
KPTN 1.34
H4C8 1.34
SCO2 1.32
CDK2 1.32
ERF 1.32
EGLN1 1.32
ATP6V0B 1.32
STIP1 1.31
SESN2 1.31
H1−2 1.31
TRIB3 1.31
ANAPC11 1.30
RPLP1 1.30
MLST8 1.29
AD/Control
c)
regulation of kinase activity
SLAMF8 2.33
HSPB1 2.04
DOK7 1.92
CAV3 1.77
PYCARD 1.76
CCNP 1.75
FGFR4 1.75
ADRA2B 1.62
APOA1 1.60
NUPR1 1.59
ZC3H12A 1.58
ITGA5 1.57
SERTAD1 1.55
TREM2 1.55
ARHGEF5 1.54
SLC11A1 1.54
CERS1 1.52
IL18 1.50
CCNI2 1.49
BIRC5 1.49
TNFRSF10A 1.48
SPINDOC 1.48
SPN 1.46
EPHA2 1.44
DVL1 1.44
HYAL2 1.41
FGR 1.41
ARID5A 1.41
TMEM119 1.41
AXIN1 1.40
RASIP1 1.40
HCLS1 1.40
ADRA2C 1.40
C5AR1 1.40
PGF 1.39
TRAF2 1.39
EPHB4 1.39
RAMP1 1.39
CDKN1A 1.38
NOS3 1.38
AD/Control
down−regulated
d)
organic acid catabolic process
TYRP1 −1.93
ALDH1A1 −1.85
NOS2 −1.77
TDO2 −1.70
DCT −1.69
KMO −1.59
DIO2 −1.58
PAH −1.57
ADTRP −1.53
ADHFE1 −1.47
ACADSB −1.47
ABCD2 −1.45
CYP4F11 −1.45
ACAD11 −1.45
ALDH7A1 −1.45
KYAT3 −1.41
AADAT −1.40
RIDA −1.40
ALDH5A1 −1.38
GAD2 −1.38
DMGDH −1.37
GLUD1 −1.37
BCKDHB −1.36
ENOSF1 −1.36
ACOT4 −1.35
CTH −1.35
MTR −1.33
ALDH6A1 −1.33
NUDT7 −1.32
AASDHPPT −1.32
HMGCLL1 −1.32
DARS1 −1.31
IDNK −1.31
PCCB −1.31
OAT −1.30
AARSD1 −1.30
SERINC1 −1.30
DLD −1.29
SLC38A1 −1.29
GLUD2 −1.29
AD/Control
e)
behavior
CRH −1.75
SLC1A2 −1.56
TPBG −1.54
TAC1 −1.49
EGR1 −1.49
DPP4 −1.48
TAS2R5 −1.43
SCN11A −1.43
GCNT4 −1.41
SLC6A1 −1.41
NTRK2 −1.40
BDNF −1.40
CHRNA7 −1.39
SNAP25 −1.38
LRRK2 −1.35
P2RY1 −1.35
PTN −1.34
S100B −1.34
ARC −1.34
KCNQ3 −1.33
PLCB1 −1.33
CNTNAP2 −1.33
SCN1A −1.32
CRHBP −1.32
NETO1 −1.31
PPT1 −1.31
PJA2 −1.30
PTPRZ1 −1.29
CSMD1 −1.29
SERPINE2 −1.28
HPRT1 −1.27
CACNB4 −1.27
PRKAA1 −1.26
ALDH1A3 −1.26
ATP1B2 −1.25
CHL1 −1.25
HTR2A −1.24
HIF1A −1.23
UBR3 −1.23
ATAD1 −1.23
AD/Control
f)
acyl−CoA metabolic process
TYRP1 −1.93
TDO2 −1.70
ACSL6 −1.44
AADAT −1.40
SUCLG2 −1.37
HSD17B12 −1.37
HSD17B8 −1.37
HMGCS1 −1.36
ACOT4 −1.35
CTH −1.35
ARSG −1.34
MTR −1.33
NUDT7 −1.32
HMGCLL1 −1.32
PPT1 −1.31
PPCS −1.30
DLD −1.29
FAR2 −1.27
HS6ST3 −1.26
AHCYL1 −1.26
DLAT −1.25
MCEE −1.25
MCCC2 −1.25
EXTL2 −1.25
HS3ST5 −1.24
AASS −1.24
SUCLA2 −1.23
PANK3 −1.22
ACSBG1 −1.22
NUBPL −1.21
NFU1 −1.21
ACSL3 −1.21
UST −1.20
ENOPH1 −1.19
CSGALNACT1 −1.19
AD/Control

Supplementary Fig. 10. Alzheimer's disease versus control Metascape gene heatmaps. Gene heatmaps comparing AD (Alzheimer's disease) samples to control samples. The heatmaps illustrate the log_2 fold change (log_2FC) values for the top 40 genes associated with selected up-regulated and down-regulated gene ontology terms within the AD versus control comparison.

a) **up–regulated**

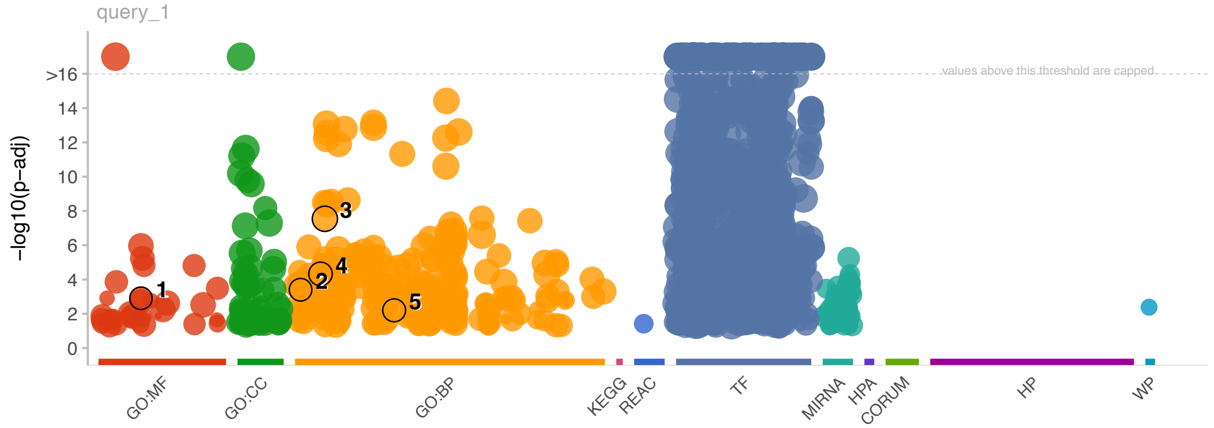

id	source	term_id	term_name	intersection_size		p_value
1	GO:MF	GO:0019901	protein kinase binding	32		1.2e−03
2	GO:BP	GO:0001816	cytokine production	97		3.9e−04
3	GO:BP	GO:0006950	response to stress	387		2.9e−08
4	GO:BP	GO:0006468	protein phosphorylation	151		4.7e−05
5	GO:BP	GO:0034097	response to cytokine	105		6.3e−03

g:Profiler (biit.cs.ut.ee/gprofiler)

b) **down–regulated**

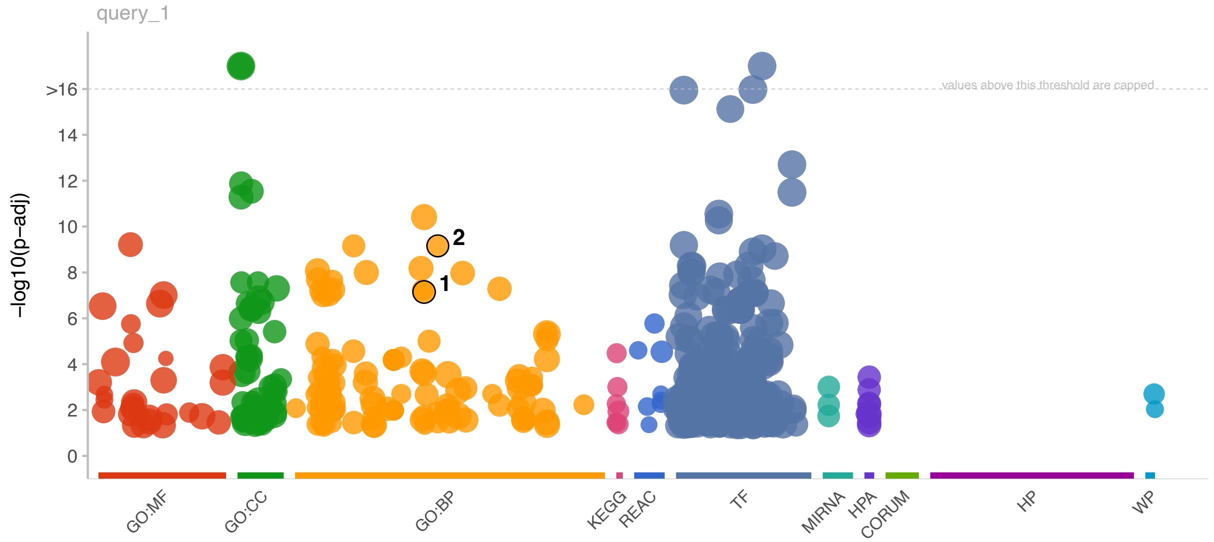

id	source	term_id	term_name	intersection_size		p_value
1	GO:BP	GO:0044282	small molecule catabolic process	28		7.1e−08
2	GO:BP	GO:0046395	carboxylic acid catabolic process	41		6.9e−10

g:Profiler (biit.cs.ut.ee/gprofiler)

Supplementary Fig. 11. Manhattan plot of Alzheimer's disease versus control g:Profiler up and down-regulated enrichment analysis. a) up-regulated and **b)** down-regulated enrichment terms. The x-axis of the plot corresponds to functional terms, which are grouped and color-coded based on data sources. The y-axis represents the adjusted enrichment -log10 *p*-values. The *p*-values are capped at less than 10^-16. The size of the circle is proportional to the size of the corresponding functional term. The table highlights selected GO (gene ontology) terms.

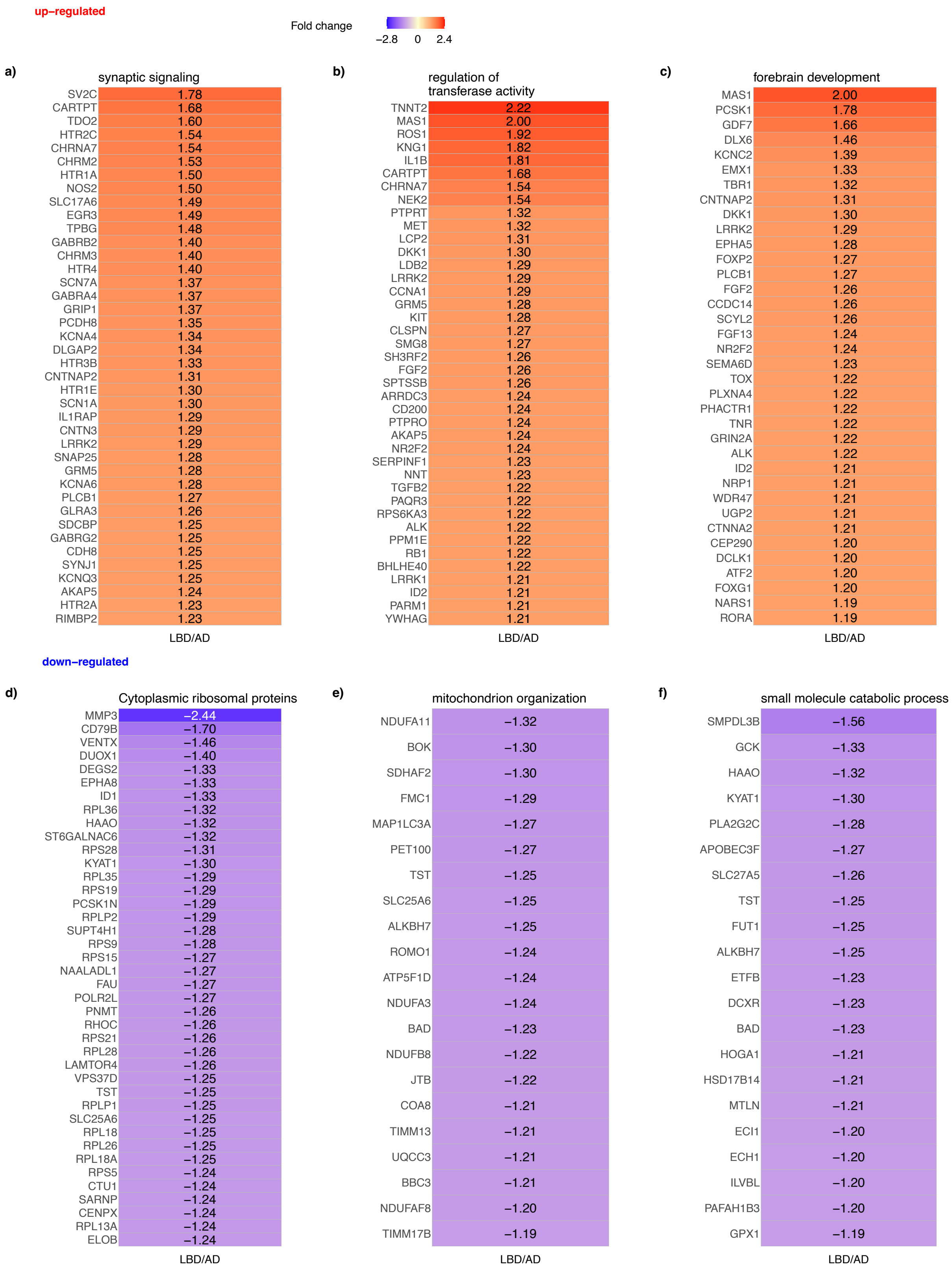

up−regulated
Fold change
−2.8 0 2.4
a)
synaptic signaling
SV2C 1.78
CARTPT 1.68
TDO2 1.60
HTR2C 1.54
CHRNA7 1.54
CHRM2 1.53
HTR1A 1.50
NOS2 1.50
SLC17A6 1.49
EGR3 1.49
TPBG 1.48
GABRB2 1.40
CHRM3 1.40
HTR4 1.40
SCN7A 1.37
GABRA4 1.37
GRIP1 1.37
PCDH8 1.35
KCNA4 1.34
DLGAP2 1.34
HTR3B 1.33
CNTNAP2 1.31
HTR1E 1.30
SCN1A 1.30
IL1RAP 1.29
CNTN3 1.29
LRRK2 1.29
SNAP25 1.28
GRM5 1.28
KCNA6 1.28
PLCB1 1.27
GLRA3 1.26
SDCBP 1.25
GABRG2 1.25
CDH8 1.25
SYNJ1 1.25
KCNQ3 1.25
AKAP5 1.24
HTR2A 1.23
RIMBP2 1.23
LBD/AD
b)
regulation of
transferase activity
TNNT2 2.22
MAS1 2.00
ROS1 1.92
KNG1 1.82
IL1B 1.81
CARTPT 1.68
CHRNA7 1.54
NEK2 1.54
PTPRT 1.32
MET 1.32
LCP2 1.31
DKK1 1.30
LDB2 1.29
LRRK2 1.29
CCNA1 1.29
GRM5 1.28
KIT 1.28
CLSPN 1.27
SMG8 1.27
SH3RF2 1.26
FGF2 1.26
SPTSSB 1.26
ARRDC3 1.24
CD200 1.24
PTPRO 1.24
AKAP5 1.24
NR2F2 1.24
SERPINF1 1.23
NNT 1.23
TGFB2 1.22
PAQR3 1.22
RPS6KA3 1.22
ALK 1.22
PPM1E 1.22
RB1 1.22
BHLHE40 1.22
LRRK1 1.21
ID2 1.21
PARM1 1.21
YWHAG 1.21
LBD/AD
c)
forebrain development
MAS1 2.00
PCSK1 1.78
GDF7 1.66
DLX6 1.46
KCNC2 1.39
EMX1 1.33
TBR1 1.32
CNTNAP2 1.31
DKK1 1.30
LRRK2 1.29
EPHA5 1.28
FOXP2 1.27
PLCB1 1.27
FGF2 1.26
CCDC14 1.26
SCYL2 1.26
FGF13 1.24
NR2F2 1.24
SEMA6D 1.23
TOX 1.22
PLXNA4 1.22
PHACTR1 1.22
TNR 1.22
GRIN2A 1.22
ALK 1.22
ID2 1.21
NRP1 1.21
WDR47 1.21
UGP2 1.21
CTNNA2 1.21
CEP290 1.20
DCLK1 1.20
ATF2 1.20
FOXG1 1.20
NARS1 1.19
RORA 1.19
LBD/AD
down−regulated
d)
Cytoplasmic ribosomal proteins
MMP3 −2.44
CD79B −1.70
VENTX −1.46
DUOX1 −1.40
DEGS2 −1.33
EPHA8 −1.33
ID1 −1.33
RPL36 −1.32
HAAO −1.32
ST6GALNAC6 −1.32
RPS28 −1.31
KYAT1 −1.30
RPL35 −1.29
RPS19 −1.29
PCSK1N −1.29
RPLP2 −1.29
SUPT4H1 −1.28
RPS9 −1.28
RPS15 −1.27
NAALADL1 −1.27
FAU −1.27
POLR2L −1.27
PNMT −1.26
RHOC −1.26
RPS21 −1.26
RPL28 −1.26
LAMTOR4 −1.26
VPS37D −1.25
TST −1.25
RPLP1 −1.25
SLC25A6 −1.25
RPL18 −1.25
RPL26 −1.25
RPL18A −1.25
RPS5 −1.24
CTU1 −1.24
SARNP −1.24
CENPX −1.24
RPL13A −1.24
ELOB −1.24
LBD/AD
e)
mitochondrion organization
NDUFA11 −1.32
BOK −1.30
SDHAF2 −1.30
FMC1 −1.29
MAP1LC3A −1.27
PET100 −1.27
TST −1.25
SLC25A6 −1.25
ALKBH7 −1.25
ROMO1 −1.24
ATP5F1D −1.24
NDUFA3 −1.24
BAD −1.23
NDUFB8 −1.22
JTB −1.22
COA8 −1.21
TIMM13 −1.21
UQCC3 −1.21
BBC3 −1.21
NDUFAF8 −1.20
TIMM17B −1.19
LBD/AD
f)
small molecule catabolic process
SMPDL3B −1.56
GCK −1.33
HAAO −1.32
KYAT1 −1.30
PLA2G2C −1.28
APOBEC3F −1.27
SLC27A5 −1.26
TST −1.25
FUT1 −1.25
ALKBH7 −1.25
ETFB −1.23
DCXR −1.23
BAD −1.23
HOGA1 −1.21
HSD17B14 −1.21
MTLN −1.21
ECI1 −1.20
ECH1 −1.20
ILVBL −1.20
PAFAH1B3 −1.20
GPX1 −1.19
LBD/AD

Supplementary Fig. 12. Lewy body disease versus Alzheimer's disease Metascape gene heatmaps. The heatmaps illustrate the $\log_2$ fold change ($\log_2$FC) values for the top 40 genes associated with selected up-regulated and down-regulated Gene Ontology (GO) terms within the Lewy body disease (LBD) versus Alzheimer's disease (AD) comparison.

a) **up–regulated**

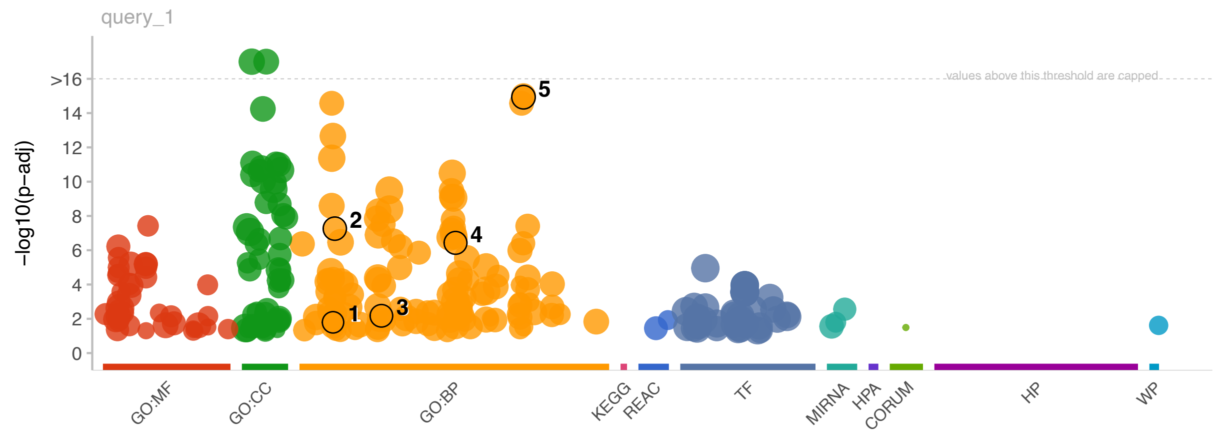

id	source	term_id	term_name	intersection_size		p_value
1	GO:BP	GO:0007416	synapse assembly	17		1.6e−02
2	GO:BP	GO:0007610	behavior	47		5.5e−08
3	GO:BP	GO:0030900	forebrain development	27		6.7e−03
4	GO:BP	GO:0050804	modulation of chemical synaptic transmission	35		3.7e−07
5	GO:BP	GO:0099536	synaptic signaling	67		1.2e−15

g:Profiler (biit.cs.ut.ee/gprofiler)

b) **down–regulated**

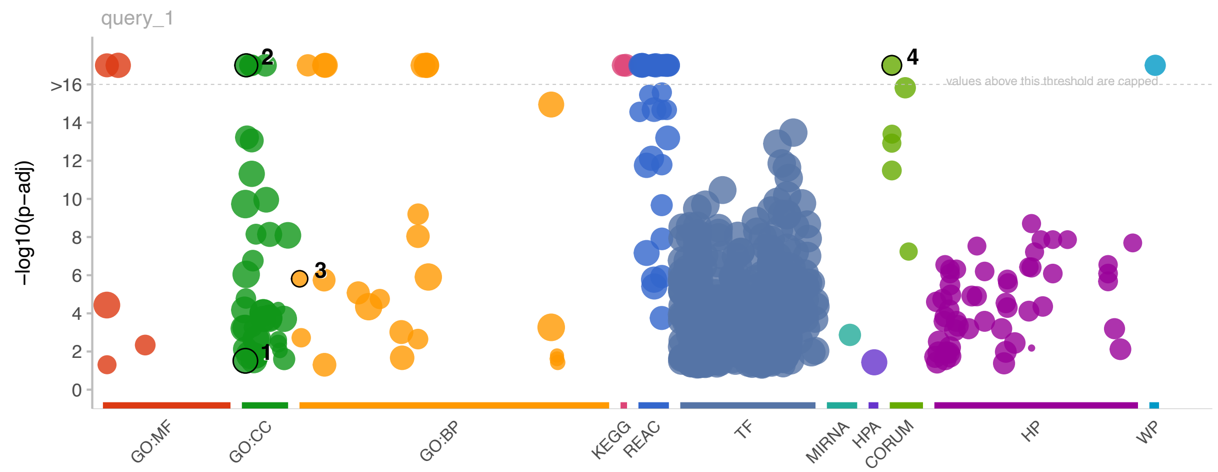

id	source	term_id	term_name	intersection_size		p_value
1	GO:CC	GO:0005739	mitochondrion	31		3.1e−02
2	GO:CC	GO:0005840	ribosome	25		2.9e−26
3	GO:BP	GO:0000028	ribosomal small subunit assembly	5		1.5e−06
4	CORUM	CORUM:306	Ribosome, cytoplasmic	24		3.6e−27

g:Profiler (biit.cs.ut.ee/gprofiler)

Supplementary Fig. 13. Manhattan plot of Lewy body disease (LBD) versus Alzheimer's disease (AD) g:Profiler up and down-regulated enrichment analysis. a) up-regulated and **b)** down-regulated enrichment terms.

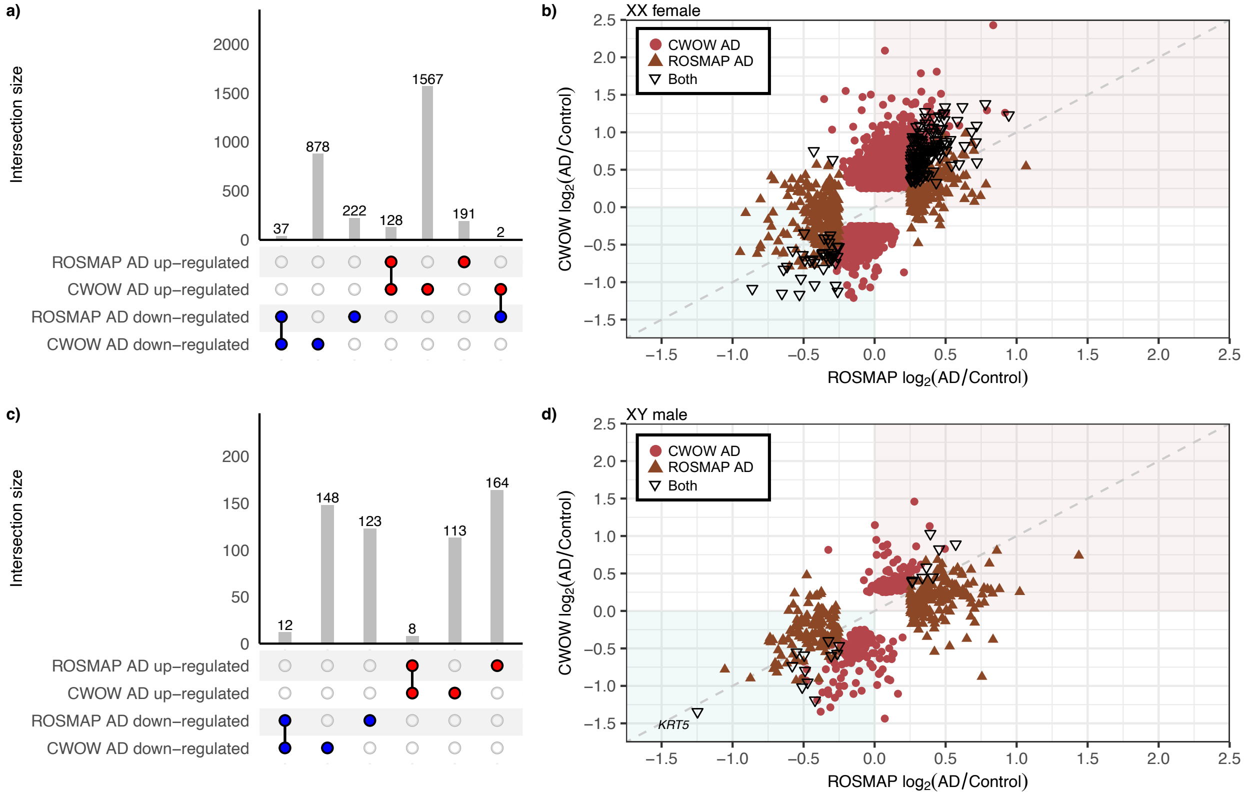

Supplementary Fig. 14. Sex-specific gene expression differences in Alzheimer's disease versus control. a) XX female only analysis UpSet plot showing the number of differentially expressed genes (q-value < 0.05 & absolute $\log_2$FC > 0.25) shared and unique between Alzheimer's disease (AD) versus control reported in this Center With Out Walls (CWOW) study and previously reported Religious Orders Study and Rush Memory and Aging Project (ROSMAP) synapse Harmonization study. **d)** XX female only analysis scatter plot of the $\log_2$FC of ROSMAP AD versus control (x-axis) and CWOW AD versus control (y-axis) reveals differentially expressed genes common to both datasets (black open triangles), unique to this CWOW dataset (maroon

circles), or unique to the ROSMAP dataset (brown triangles). Genes within the bottom left teal box had a negative log_2FC in both datasets and genes within the top right pink box had a positive log_2FC in both. The above repeated for XY male only analysis **c)** UpSet and **d)** scatter plot.

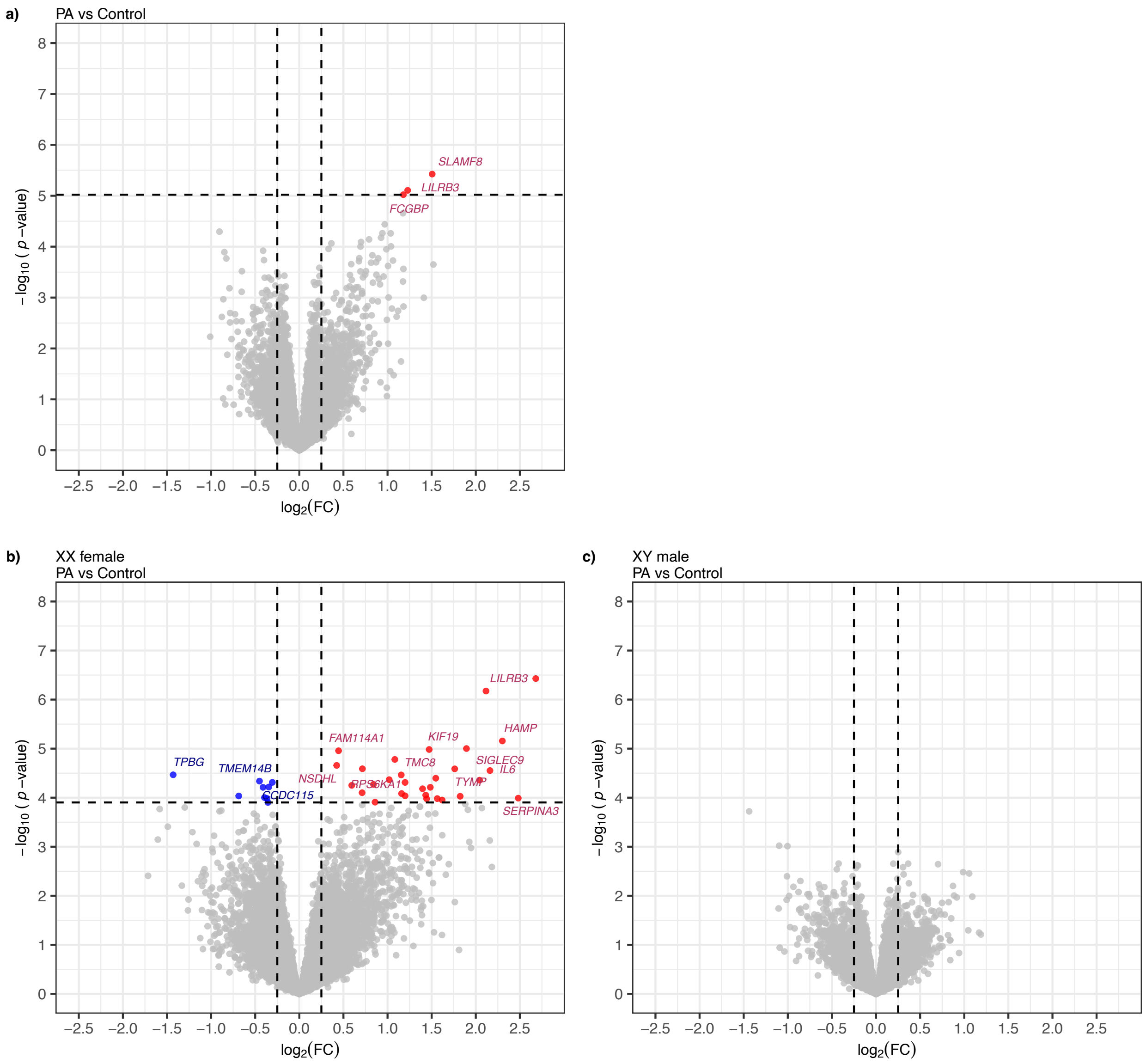

Supplementary Fig. 15. Gene differential expression analysis in pathological amyloid versus control. a) Gene differential analysis between predominant amyloid-only pathology (PA, *n* = 39) compared to controls (*n* = 81) resulted in only three significantly differentially expressed genes,

q-value < 0.05 & absolute $\log_2 FC > 0.25$. **b)** Gene differential expression between XX female PA (n = 16) versus XX female control (n = 31) resulted in 39 differentially expressed genes (DEGs). **c)** Gene differential expression between XY male PA (n = 23) versus XY male control (n = 50) resulted in no DEGs.

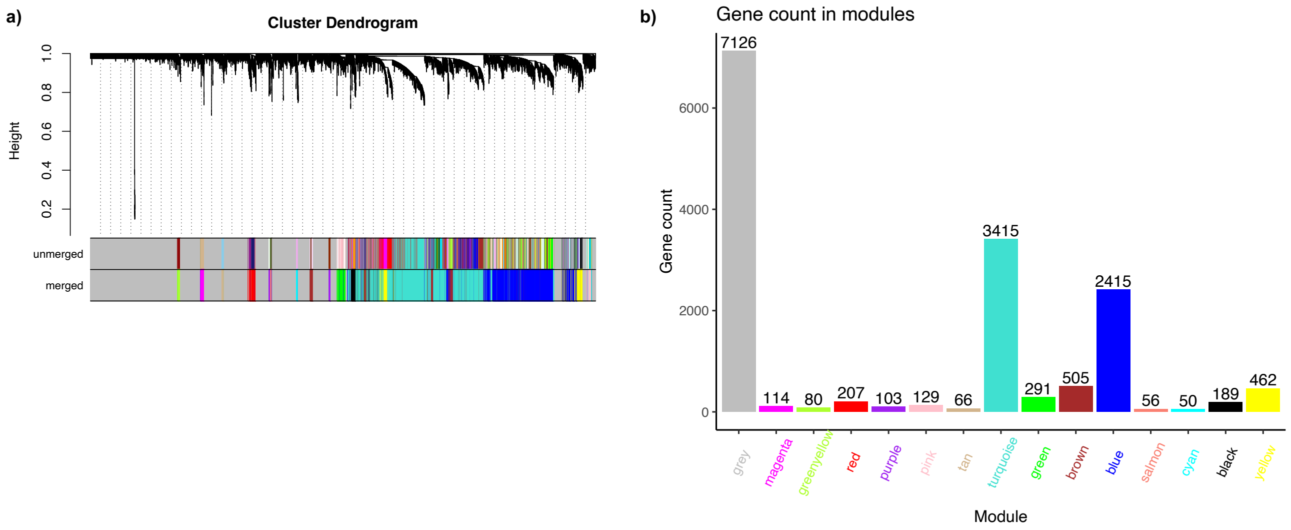

Supplementary Fig. 16. Weighted Gene Correlation Network Analysis (WGCNA) dendrogram and gene count within each module. a) Cluster dendrogram based on the topological overlap of gene expression across all samples (n = 609). The colored panels beneath the dendrogram show unmerged (top) and merged (bottom) modules obtained from WGCNA. **b)** Gene count for each merged gene set module

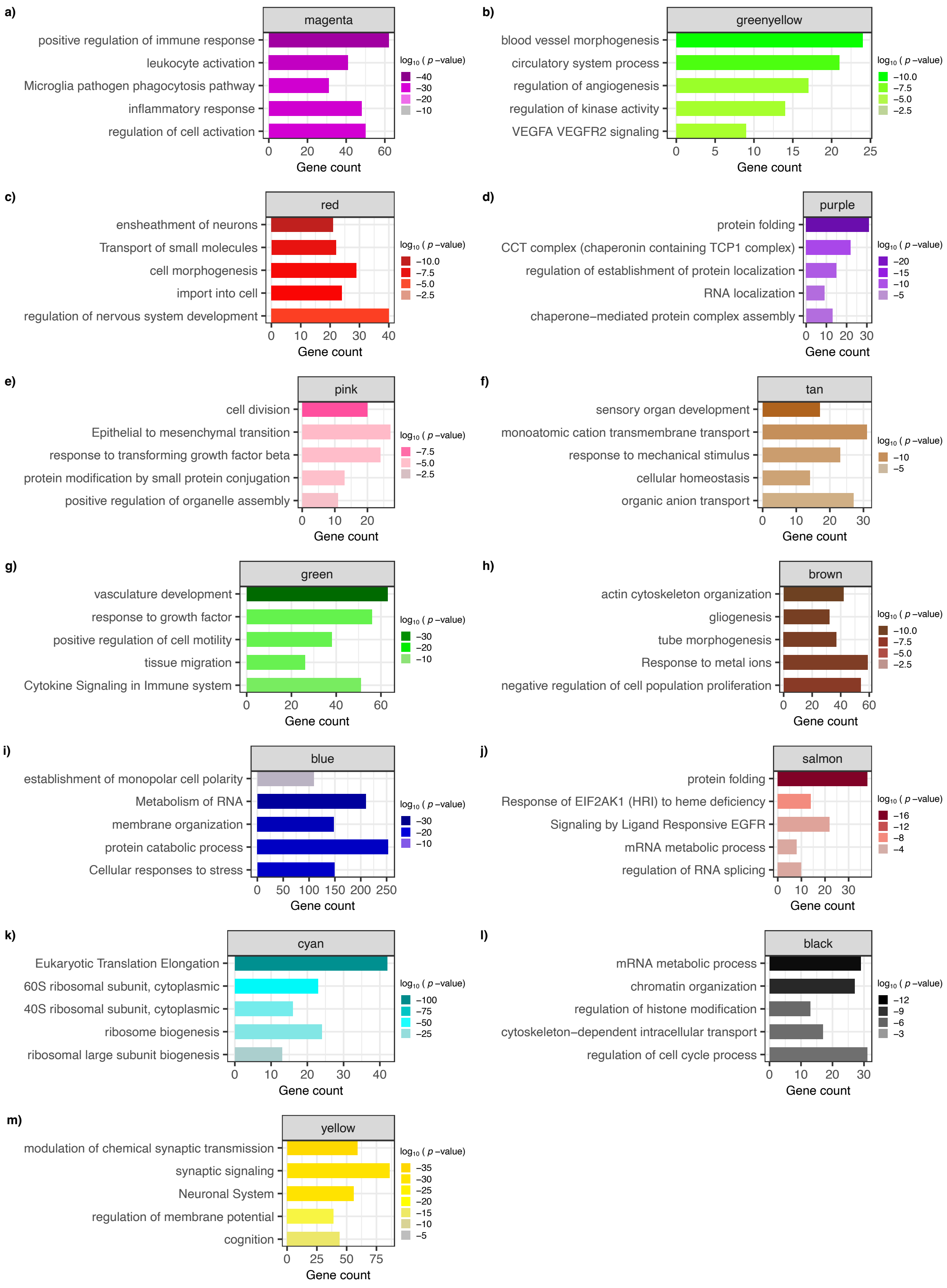

a)
magenta
positive regulation of immune response
leukocyte activation
Microglia pathogen phagocytosis pathway
inflammatory response
regulation of cell activation
Gene count
log10 (p −value)
b)
greenyellow
blood vessel morphogenesis
circulatory system process
regulation of angiogenesis
regulation of kinase activity
VEGFA VEGFR2 signaling
c)
red
ensheathment of neurons
Transport of small molecules
cell morphogenesis
import into cell
regulation of nervous system development
d)
purple
protein folding
CCT complex (chaperonin containing TCP1 complex)
regulation of establishment of protein localization
RNA localization
chaperone−mediated protein complex assembly
e)
pink
cell division
Epithelial to mesenchymal transition
response to transforming growth factor beta
protein modification by small protein conjugation
positive regulation of organelle assembly
f)
tan
sensory organ development
monoatomic cation transmembrane transport
response to mechanical stimulus
cellular homeostasis
organic anion transport
g)
green
vasculature development
response to growth factor
positive regulation of cell motility
tissue migration
Cytokine Signaling in Immune system
h)
brown
actin cytoskeleton organization
gliogenesis
tube morphogenesis
Response to metal ions
negative regulation of cell population proliferation
i)
blue
establishment of monopolar cell polarity
Metabolism of RNA
membrane organization
protein catabolic process
Cellular responses to stress
j)
salmon
protein folding
Response of EIF2AK1 (HRI) to heme deficiency
Signaling by Ligand Responsive EGFR
mRNA metabolic process
regulation of RNA splicing
k)
cyan
Eukaryotic Translation Elongation
60S ribosomal subunit, cytoplasmic
40S ribosomal subunit, cytoplasmic
ribosome biogenesis
ribosomal large subunit biogenesis
l)
black
mRNA metabolic process
chromatin organization
regulation of histone modification
cytoskeleton−dependent intracellular transport
regulation of cell cycle process
m)
yellow
modulation of chemical synaptic transmission
synaptic signaling
Neuronal System
regulation of membrane potential
cognition

Supplementary Fig. 17. Gene ontology (GO) enrichment summaries for Weighted Gene Correlation Network Analysis gene modules. Overview of the top five Gene Ontology (GO) enrichment summaries for the Weighted Gene Correlation Network Analysis (WGCNA) gene modules. Metascape was employed to elucidate the biological and functional significance each gene module. Two gene modules, namely "gray" and "turquoise," are not shown because these particular gene modules contain an excess of 3,000 genes, surpassing the maximum allowable limit for Metascape GO analysis.

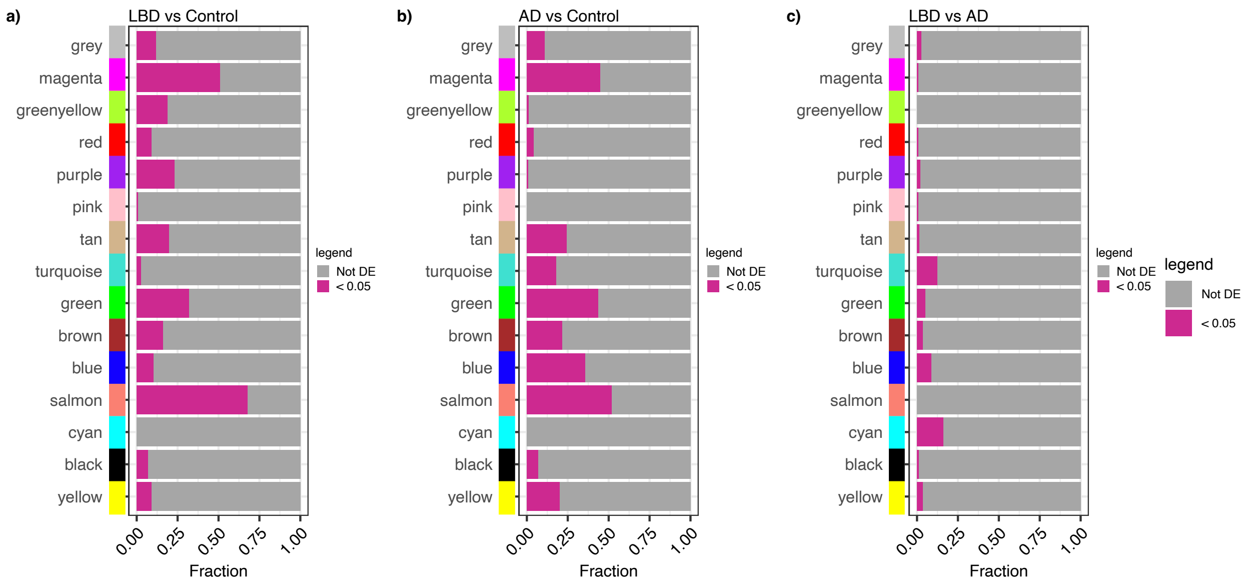

Supplementary Fig. 18. Fraction of genes within Weighted Gene Correlation Network Analysis (WGCNA) gene modules identified as differentially expressed. a) Lewy body disease (LBD) versus Control, **b)** Alzheimer's disease (AD) versus Control, and **c)** LBD versus AD.
